# Benchmarking the PAM compatibility of Cas12a variants for high-throughput yeast genetic variant engineering

**DOI:** 10.1128/aem.01618-25

**Published:** 2025-11-25

**Authors:** Weiyu Xie, Zhenkun Cai, Zehua Bao

**Affiliations:** 1 Key Laboratory of Biomass Chemical Engineering of Ministry of Education, College of Chemical and Biological Engineering, Zhejiang University 12377, Hangzhou, Zhejiang, China; 2 Zhejiang Key Laboratory of Intelligent Manufacturing for Functional Chemicals, ZJU-Hangzhou Global Scientific and Technological Innovation Center, Zhejiang University 12377, Hangzhou, Zhejiang, China; 3 Institute of Bioengineering, College of Chemical and Biological Engineering, Zhejiang University 12377, Hangzhou, Zhejiang, China; 4 Zhejiang Key Laboratory of Smart Biomaterials, College of Chemical and Biological Engineering, Zhejiang University 12377, Hangzhou, Zhejiang, China; Chalmers tekniska hogskola AB, Gothenburg, Sweden

**Keywords:** Cas12a, *Saccharomyces cerevisiae*, CRISPR, gene editing

## Abstract

*Saccharomyces cerevisiae* is an important organism for basic research and applied biotechnology. Genome editing techniques, particularly CRISPR/Cas9 from *Streptococcus pyogenes*, have greatly facilitated saturation genome editing in yeast to understand mutant functions on a large scale. However, Cas9 is restricted by its targeting preference for G-rich protospacer-adjacent motif (PAM) sequences. To broaden the targeting scope, we established an efficient homology-integrated CRISPR/Cas12a system to install genetic variants through homologous recombination by targeting T-rich PAMs. We benchmarked the PAM compatibility of PAM-relaxed Cas12a variants and identified the improved LbCas12a (impLbCas12a) as the most efficient and PAM-relaxed variant in *S. cerevisiae*, showing high editing purity and an editing window centering the double-strand break. We show that our system can be used to perform targeted saturation mutagenesis to reveal functional variants not captured previously. By using a homology-integrated CRISPR RNA array, we utilized the multiplexing capability of CRISPR/Cas12a to realize multiplex genetic variant installation. Our system enriches the yeast genetic variant engineering toolbox, complementing the commonly used CRISPR/Cas9 system.

**IMPORTANCE:**

CRISPR/Cas9 has facilitated yeast functional genomics by generating a large number of precise genetic variants in a very short period of time. This enabled the interrogation of reconstituted natural genetic variants across different genetic backgrounds or entirely synthetic mutations to discover novel or improved functions. However, Cas9 only targets a limited genomic sequence space due to its preference for G-rich PAM sequences. In this study, we close this gap by developing a CRISPR/Cas12a-based system to engineer user-defined genetic variants targeting T-rich PAM sequences. Our system adopts a homology-integrated design and the most PAM-relaxed Cas12a characterized in yeast to date. These features collectively enabled the creation of genetic variant libraries and multiplex edited strains. This genome editing tool can be used together with Cas9-based tools to interrogate a greater genomic sequence space.

## INTRODUCTION

As a model eukaryote, yeast (*Saccharomyces cerevisiae*) is extensively studied to investigate various eukaryotic biological processes. Studies revealed an average of 32% amino acid consensus between *S. cerevisiae* and human across the protein-coding genome (1). Nearly 30% of known human disease-related genes have yeast homologs (2). Hence, mutational studies in yeast help us understand human biology and disease mechanisms (3). Besides its vast utility in basic research, yeast is also used as a chassis organism in a wide range of industrial applications (4), including enzyme and microbial cell factory engineering. Being able to engineer precise genetic variants in yeast thus facilitates both basic and applied research. Yeast offers a trackable genetic background with a complete genome sequence (5). Its inherently efficient homologous recombination (HR) machinery facilitated precision genetic engineering in this species (6). Particularly, with the application of CRISPR/Cas systems in *S. cerevisiae*, especially the type II CRISPR/Cas9 system from *Streptococcus pyogenes*, genetic variant engineering in yeast has become routine (7–9). In a typical CRISPR-based genetic variant engineering protocol, a guide RNA (gRNA) and a HR donor were co-delivered into the yeast cell. The *Streptococcus pyogenes* Cas9 (SpCas9) was targeted by the gRNA to a sequence-matched protospacer, which precedes a protospacer-adjacent motif (PAM). SpCas9 then cleaves the DNA at approximately three nucleotides 5′ of the PAM to generate a blunt-end double-strand break, which is repaired through HR with the supplied donor. Genetic variants encoded in the HR donor are thus integrated in a seamless manner. By scaling up this approach, high-throughput variant libraries can be created rapidly for functional genomics and directed evolution (10).

A major limitation of SpCas9 is that it recognizes the G-rich PAM in the form of NGG, which occurs infrequently in the yeast genome with a 61.5% AT content (GenBank: GCA_000146045.1). While gene knockout is less constrained by the scarcity of NGG PAMs, it becomes a concern when performing genetic variant engineering, since only a handful of PAMs near the intended edit are available. In addition, not all the gRNAs targeting these PAMs will be specific and efficient, further reducing the choices available. Although several PAM-relaxed SpCas9 variants were developed (11–13), our previous study found that the most-relaxed variant SpG and SpRY both suffer from reduced editing efficiencies as compared to SpCas9 (14). The more efficient variant SpG still prefers non-AT PAMs.

The CRISPR/Cas12a system (formerly known as CRISPR/Cpf1), which recognizes a T-rich PAM, complements SpCas9 in PAM preferences (15). Cas12a possesses unique features that distinguish it from SpCas9. First, Cas12a is guided by a CRISPR RNA (crRNA) that is structurally simpler and shorter than SpCas9 gRNA; second, Cas12a cleaves at the distal end of the PAM sequence, producing sticky ends that may enhance homology-directed repair (HDR) efficiency in comparison to blunt ends (16, 17); third, Cas12a is able to process the precursor crRNA array on its own (18), rendering it an advantage in multiplex gene editing (19), while extra genetic parts such as ribozymes and tRNAs are typically required for SpCas9 gRNA array processing (20). All these features make Cas12a a promising nuclease for high-throughput and multiplex variant engineering, where simplicity is highly desired to facilitate crRNA design, synthesis, and cloning.

Cas12a orthologs have been used to perform genome editing in *S. cerevisiae* as well as in other yeast species (21–26). In *S. cerevisiae*, Cas12a from three species (LbCas12a, AsCas12a, and FnCas12a) was employed to integrate a yellow fluorescent protein gene and a three-gene carotenoid synthesis pathway (21). LbCas12a and FnCas12a exhibited editing efficiencies similar to SpCas9, while AsCas12a showed a lower efficiency. In addition, LbCas12a was shown to enable multiplex heterologous gene integration using a crRNA array. In another study, FnCas12a facilitated stop codon installation and multiplex gene knockout using co-transformed linear double-strand DNA as repair donors, achieving efficiencies of up to 100% (22). However, more advanced use of Cas12a in yeast genetic variant engineering, particularly high-throughput and multiplex codon replacement, has not been realized. One technical issue is that linear repair donors are not compatible with high-throughput genetic variant engineering, in which the donor must be physically paired with the crRNA to ensure co-delivery in a complex pooled library (27–31).

The other limitation is that the targeting range of most wild-type Cas12a orthologs is constrained by their long PAM sequences (TTTV, where V = A, C, G). To solve this problem, researchers have developed PAM-relaxed Cas12a variants and applied them in mammalian cells. Through structure-guided engineering, RR (S542R/K607R) and RVR (S542R/K548V/N552R) variants of AsCas12a were obtained, recognizing TYCV and TATV PAMs, respectively (32). By introducing similar mutations, LbCas12a-RR and LbCas12a-RVR can recognize TYYV and TWTV, respectively. enAsCas12a, which contained E174R, S542R, and K548R mutations, can recognize TTYN (Y = T, C), VTTV, and TRTV (R = A, G) (33). This variant expands the targeting range of the wild-type AsCas12a by about sevenfold. By combining the above effective mutations into LbCas12a, the impLbCas12a variant (D156R/G532R/K538V/Y542R/K595R) can recognize TNTN PAMs as well as TACV, TTCV, TCCV, CTCV, and CCCV PAMs in mammalian cells (34). Similarly, through structure-guided engineering, six mutations (K180S, N607R, K613V, D616N, N617R, and K660R) were introduced into *Francisella novicida* Cas12a (FnCas12a) to derive the FnCas12a-EP16 variant (35). This variant was reported to recognize NNYN and NTAC PAMs *in vitro*. The PAM compatibility of these Cas12a variants, however, has not been validated in yeast.

In this study, to realize Cas12a-assisted genetic variant engineering in yeast, we first developed a homology-integrated CRISPR/Cas12a system that facilitates high-efficiency codon replacement in *S. cerevisiae* using a single plasmid, in which the HR donor is placed 3′ of the crRNA spacer sequence. Using this system, we benchmarked the codon replacement efficiencies and PAM compatibility of several PAM-relaxed Cas12a variants for yeast genetic variant engineering. We found that the impLbCas12a variant exhibited the broadest PAM compatibility with a high codon replacement accuracy. The editing efficiency and editing range of impLbCas12a at canonical PAMs are on par with the wild-type LbCas12a. Using this homology-integrated impLbCas12a system, we successfully realized high-throughput and multiplex genetic variant engineering in *S. cerevisiae*. Our approach complements SpCas9-based approaches by targeting T-rich PAMs and should facilitate genetic variant engineering in yeast for both basic and applied research.

## RESULTS

### A homology-integrated CRISPR/Cas12a system facilitates high-efficiency codon replacement in *S. cerevisiae*

**Fig 1:**
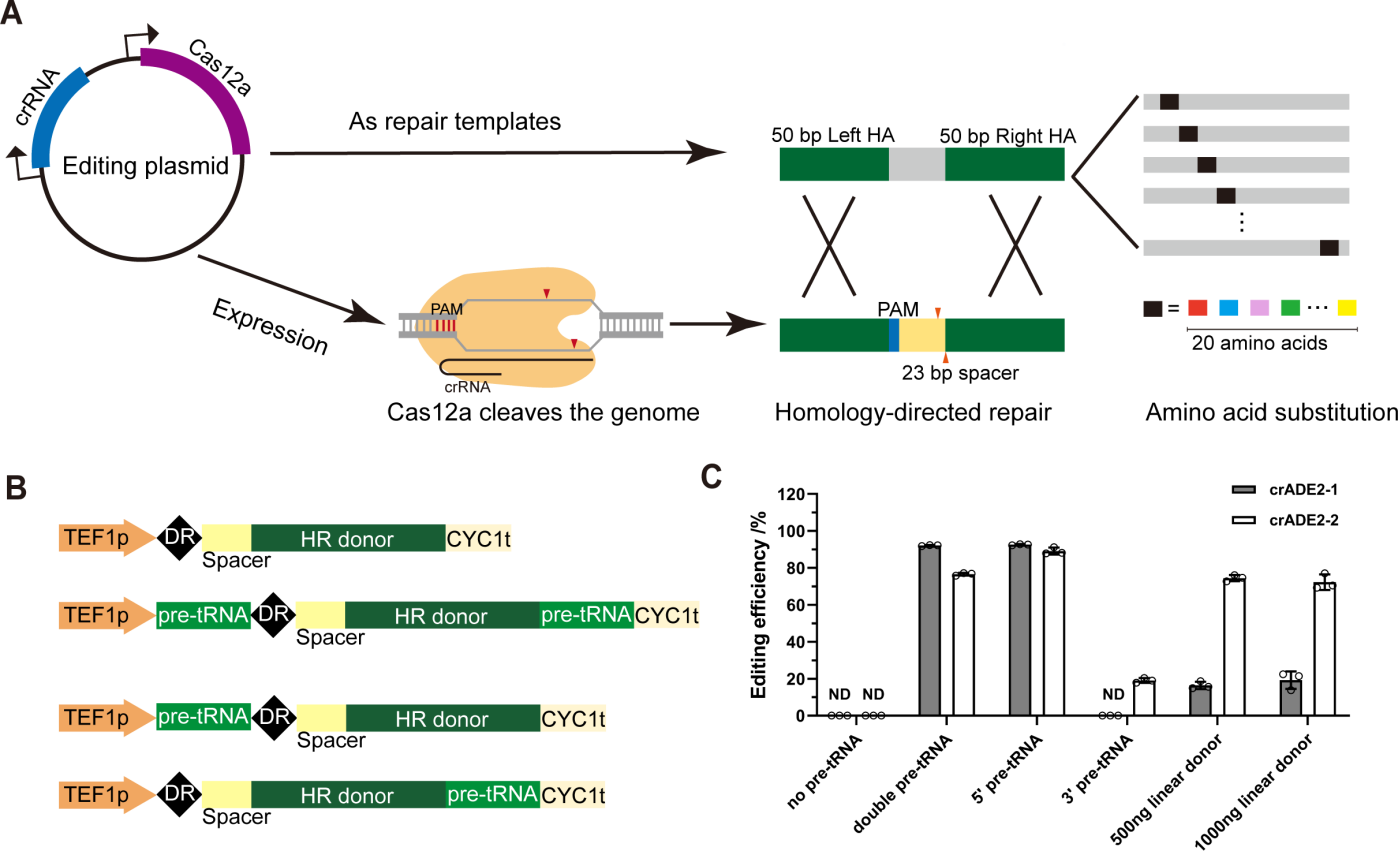
Schematic of the Cas12a-based genetic variant editing system and optimization of the crRNA expression cassette. (**A**) A single editing plasmid expresses the Cas12a nuclease, the crRNA, and harbors the repair donor. The Cas12a nuclease cleaves the crRNA-targeted site. Subsequently, the cleaved site is repaired by the donor present on the plasmid through homology-directed repair. This approach enables modifications to any of the 20 amino acids at specified codon loci. (**B**) Design of four crRNA expression cassettes with varying pre-tRNA numbers and positions. (**C**) Editing efficiencies of different cassettes using the wild-type LbCas12a and two crRNAs targeting the *ADE2* gene. The 5′ pre-tRNA design with linear donor of two different transformed amounts was used as non-integrated control. ND, not detected. *n* = 3 biological replicates. Error bars represent standard deviations.

Since current Cas12a-based yeast gene editing systems are not scalable, we first aimed to develop an efficient genetic variant engineering system with plasmid HR donors instead of linear HR donors (Fig. 1A). The Cas12a ortholog from *Lachnospiraceae bacterium* ND2006 (LbCas12a), which showed a high DNA cutting activity in *S. cerevisiae* (21), was utilized for initial testing of the system. The crRNA consists of a 20-nucleotide direct repeat (DR) sequence of the LbCas12a system and a 23-nucleotide spacer sequence. Following the design of our previously developed SpCas9-based HI-CRISPR system (9), we placed the HR donor to the 3′ end of the spacer (Fig. 1B). The donor consists of 50 bp homology arms flanking the amino acid substitution mutations, which were integrated to replace wild-type codons after Cas12a cleaving the DNA and homology directed repair (Fig. 1A). This homology-integrated CRISPR/Cas12a (HI-Cas12a) system enables simple design, synthesis, and cloning, facilitating scaling up. The entire system is expressed from a single plasmid (Fig. S1).

Considering that Pol III promoter-driven crRNA expression may be terminated by T-rich sequences (36), we opted to use a Pol II promoter TEF1p to drive crRNA expression (Fig. 1B). To prevent misprocessing of the crRNA as a messenger RNA, we placed a tRNA precursor pre-tRNA^Gly^ (37) after the promoter and/or before the terminator to facilitate crRNA cleavage from regulatory sequences (Fig. 1B). Two crRNAs targeting *ADE2* were designed for testing (Fig. S2). The donors were designed to introduce a TAA stop codon while disrupting the PAM sequence. Results indicated that only the 5′ pre-tRNA was necessary for editing, achieving up to 95% efficiency, and there was no significant difference in editing efficiencies between using 5′ pre-tRNA or both pre-tRNAs. In addition, the integrated donor design achieved higher editing efficiencies than using a linear donor (Fig. 1C). Considering that two repetitive pre-tRNA sequences may complicate cloning, we proceeded with the 5′ pre-tRNA design.

To assess editing accuracy, red colonies were randomly selected for sequencing, showing a high editing accuracy and purity (Fig. S2). The term “editing purity” denotes the proportion of correctly edited alleles. In addition, the impact of an additional duration of liquid cultivation after plasmid transformation on editing efficiency was evaluated. Higher editing efficiencies were observed without liquid cultivation (Fig. S3). Subsequent outgrowth led to a decrease in editing efficiency, which may be attributed to the growth advantage of non-edited wild-type yeast cells, especially when targeting *ADE2*, whose deletion is known to reduce cellular fitness and lifespan of *S. cerevisiae* (38). This contrasts with SpCas9-mediated editing, where further liquid cultivation gradually increased editing efficiency (9).

### Characterizing the PAM compatibility of Cas12a variants in *S. cerevisiae*

**Fig 2:**
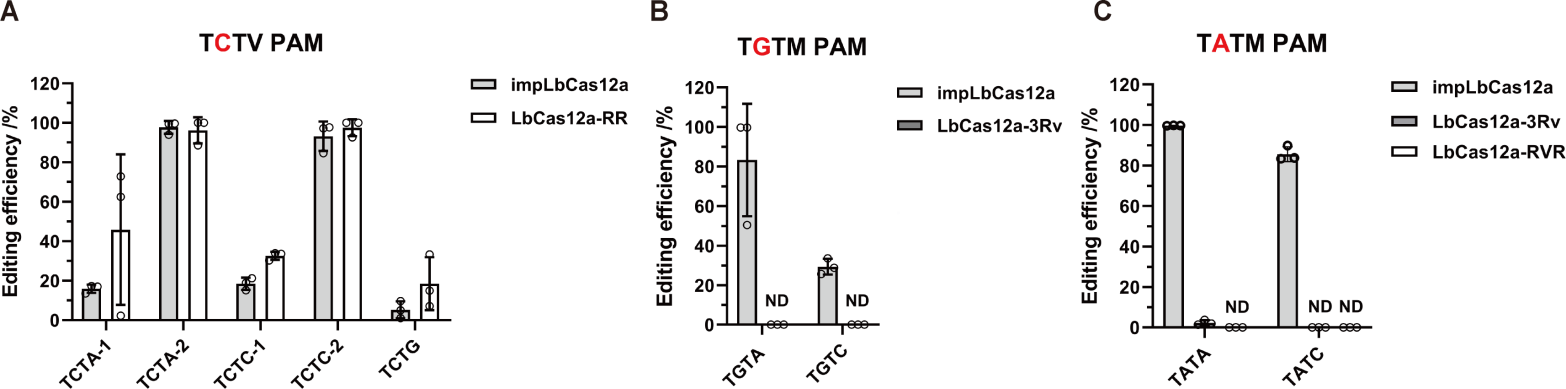
Editing efficiencies of PAM-relaxed LbCas12a variants at non-canonical PAMs. (**A**) Benchmarking impLbCas12a and LbCas12a-RR at TCTV PAMs. For the TCTA and TCTC PAM, two crRNAs were chosen for testing. (**B**) Benchmarking impLbCas12a and LbCas12a-3RV at TGTM PAMs. (**C**) Benchmarking impLbCas12a, LbCas12a-3RV, and LbCas12a-RVR at TATM PAMs. N = A, T, C, G; V = A, C, G; M = A or C. ND, not detected. *n* = 3 biological replicates. Error bars represent standard deviations.

To identify a Cas12a variant with the most flexible PAM and high on-target activity, we systematically evaluated the PAM compatibility of PAM-relaxed Cas12a variants in *S. cerevisiae*. We focused our assessment on four LbCas12a variants, namely LbCas12a-RR, LbCas12a-RVR (32), LbCas12a-3Rv (33) and impLbCas12a (34) (Fig. S4), since LbCas12a was previously shown to be more efficient than AsCas12a in yeast (21). We tested subsets of non-canonical PAMs previously reported to be functional in introducing indels in mammalian cells (Table S1). The results with impLbCas12a showed that without liquid cultivation, most of the colonies were chimeric at a non-canonical TGTC PAM (Fig. S5A). A period of liquid cultivation was necessary to obtain pure editing at several non-canonical PAMs (Fig. S5B). In light of this, we included a 5-day period of liquid cultivation after yeast transformation in subsequent PAM characterization of Cas12a variants before assessing the editing efficiency. By comparing the editing efficiencies of impLbCas12a and LbCas12a-RR at TCTV PAMs, we found that impLbCas12a exhibited comparable or slightly lower editing efficiencies than LbCas12a-RR (Fig. 2A). At TGTM PAMs, impLbCas12a edited efficiently, whereas LbCas12a-3Rv failed to recognize these PAMs (Fig. 2B). At TATM PAMs, impLbCas12a achieved up to 100% efficiency. In contrast, neither LbCas12a-RVR nor LbCas12a-3Rv could edit (Fig. 2C). However, LbCas12a-3Rv exhibited high editing efficiencies at ATTA and CTTA PAMs (Fig. S6), which are not recognizable by impLbCas12a (34). Besides LbCas12a variants, we also tested FnCas12a-EP16 (35), an *in vitro* validated PAM-relaxed variant of FnCas12a, and PrCas12a-3Rv (39) (Table S4). However, their editing efficiencies were negligible (Supplementary Text; Fig. S7 and S8). These benchmarking experiments indicated that, except for VTTV PAMs which were efficiently targeted by LbCas12a-3Rv, impLbCas12a exhibited the broadest PAM compatibility and robust genetic variant engineering efficiency in *S. cerevisiae*.

### PAM compatibility and editing purity of impLbCas12a

**Fig 3:**
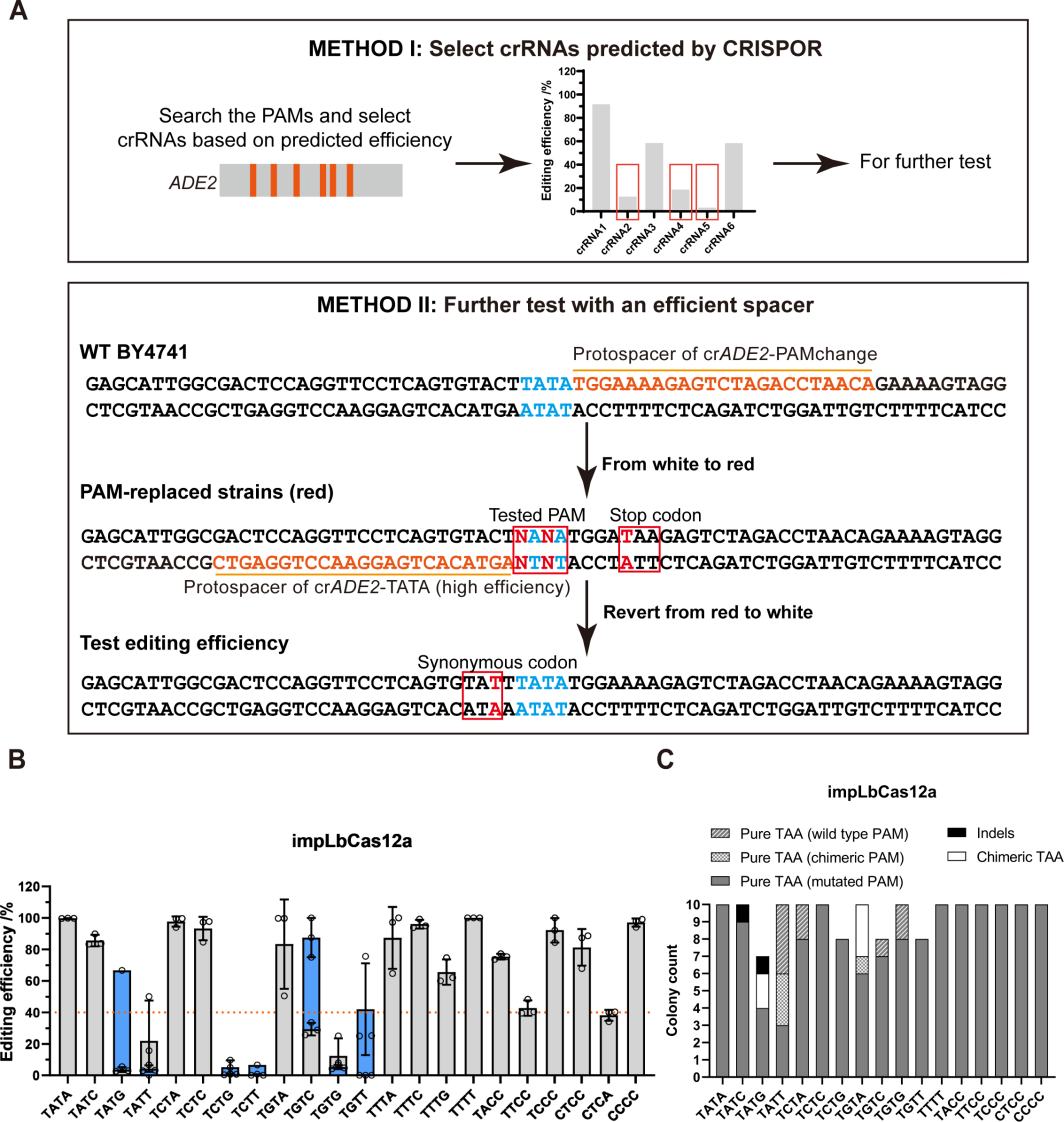
Editing efficiencies and accuracy of impLbCas12a at TNTN and C-rich PAMs. (**A**) Approach to PAM compatibility testing. Method I: screening of functional PAMs using predicted crRNAs. Method II: validation of low-efficiency PAMs from Method I using a validated high-efficiency guide sequence. Method II involves two steps. First, a guide sequence near a high-efficiency guide sequence is used to install the tested PAM while introducing a TAA stop codon to generate red PAM-replaced strains. Second, the high-efficiency crRNA is transformed into the PAM-replaced strains to recover the wild-type protein sequence and generate white colonies. Editing efficiencies were evaluated by the percentage of reverted colonies. (**B**) Editing efficiencies of impLbCas12a at all TNTN and C-rich PAMs. The blue columns represent efficiencies derived from Method II. (**C**) Genotyping of red colonies for the indicated PAMs. *n* = 3 biological replicates. Error bars represent standard deviations.

We then proceeded to comprehensively assess the PAM compatibility of impLbCas12a at TNTN and C-rich PAMs. We first screened efficient PAMs using crRNAs designed by CRISPOR (40). For PAMs showing low editing efficiencies, we further tested them with an experimentally validated high-efficiency spacer (Fig. 3A). The spacer of crADE2-TATA (Table S3) was chosen as the efficient spacer. To replace the original TATA PAM of this spacer with non-canonical PAMs to be tested, an adjacent crRNA targeting the palindromic TATA PAM on the opposite strand was used. The associated donor was designed to concurrently introduce the test PAM and a TAA stop codon. A series of red-colored PAM-replaced strains were constructed. Subsequently, crRNA-TATA was transformed into these PAM-replaced strains with a donor correcting the reading frame. Editing efficiencies at test PAMs were assessed by the percentage of colonies reverting to a white color. By combining results from both testing methods, we found that impLbCas12a effectively recognized TNTN PAMs with over 60% editing efficiency, except for TVTT and TSTG (S = C/G) PAMs. In addition, it also recognized C-rich PAMs, including TACC, TTCC, TCCC, CTCC, CTCA, and CCCC (Fig. 3B). These results largely agree with the previous study in mammalian cells (34). To evaluate editing accuracy and purity, randomly selected edited colonies were sequenced (Fig. 3C). A portion of edited colonies contained the TAA stop codon but no PAM mutation, which was originally designed as a synonymous mutation to prevent repeated Cas12a cleavage. This may be due to the longer distance between the cleavage site and the synonymous mutation in certain HR donors. Overall, impLbCas12a introduced very few indels while achieving over 40% editing efficiencies at 18 canonical and non-canonical PAMs, with over 80% efficiencies at 12 of them, affirming its superiority as a genetic variant engineering tool in yeast. This impLbCas12a editing system was also efficient in introducing stop codons into *LYP1* and *CAN1* genes (Fig. S9) and in performing integration at the promoters of several other endogenous genes of yeast (Fig. S10) by targeting both canonical and non-canonical PAMs (Table S3), suggesting potential for the genome-wide utility of this system. The editing purity of impLbCas12a was also higher than that of LbCas12a-RR and LbCas12a-3Rv (Fig. S11).

### Editing window of impLbCas12a

**Fig 4:**
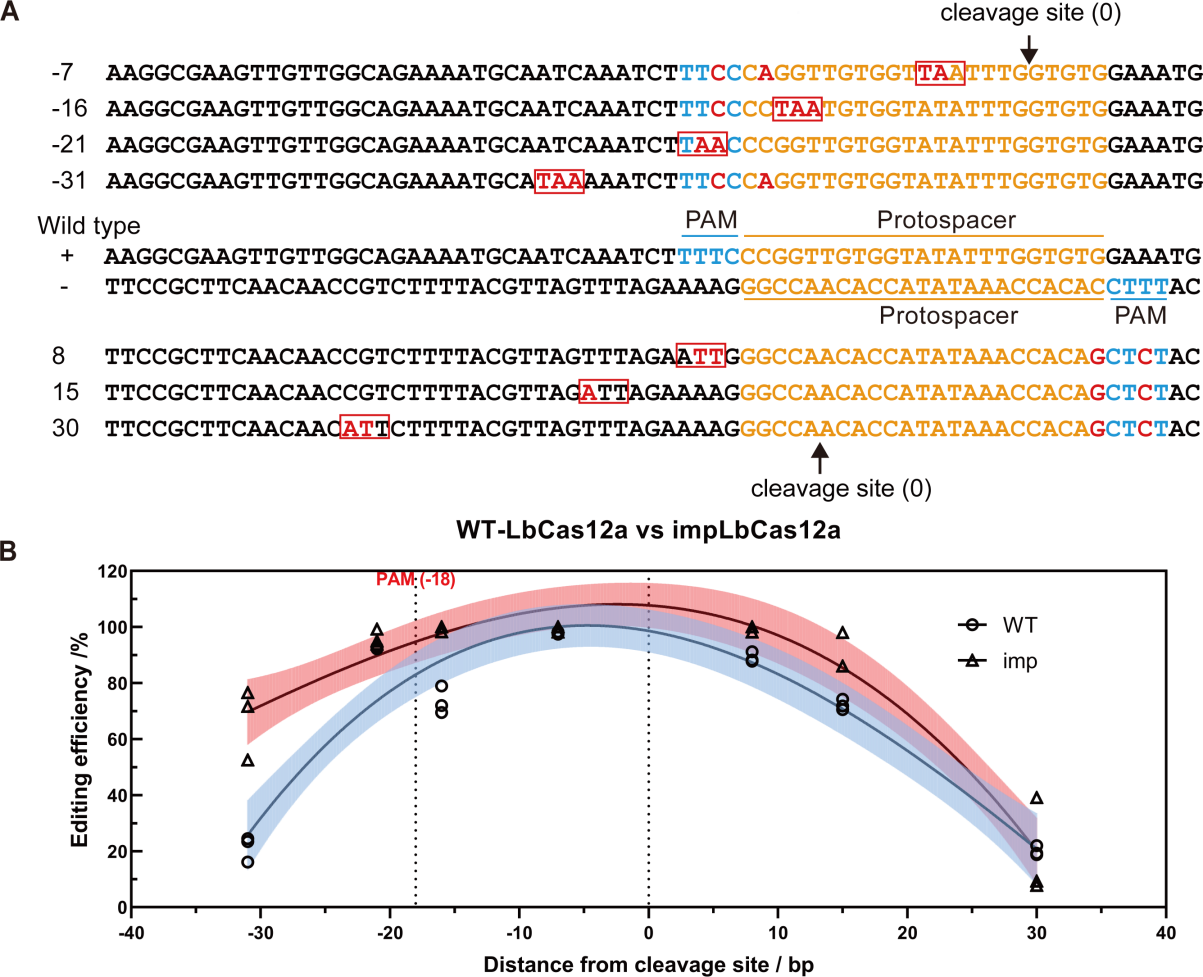
Editing range of Cas12a. (**A**) Design of donors with varying distances between the target codon and the cleavage site. Sequences in orange represent the two protospacers targeted. PAMs are highlighted in blue. Edits are highlighted in red and TAA stop codons additionally boxed. The distance in base pairs between each TAA edit and the cleavage site is denoted on the left of each donor sequence. (**B**) Relationship between editing efficiencies and editing distances of WT-LbCas12a and impLbCas12a. *n* = 3 biological replicates. The fitting was a third-order polynomial (cubic) with 95% confidence intervals.

The editing efficiency tends to drop with an increasing distance from the PAM or cleavage site in SpCas9-based approaches (27). To determine the efficient editing range of impLbCas12a using a single crRNA, we explored the relationship between editing efficiencies and positions of replaced codons. Additionally, we designed synonymous mutations within and/or close to the PAM to prevent impLbCas12a from cleaving the HR donor and the edited sequence (Fig. 4A). The distance between the PAM and the cleavage site of LbCas12a is 18 bp (Fig. 4A), in contrast to a distance of 3 bp for SpCas9. Hence, we investigated whether the editing efficiency will be the highest close to the PAM or the cleavage site. To maximally reduce variations associated with sequences and locations of spacer sequences, we designed HR donors with varying distances using the same spacer. We chose two reverse-complementary spacers to account for targeting both sense and non-sense strands (Fig. 4A). Our findings revealed that the editing efficiency increased as the intended TAA codon replacement approaches the cleavage site (Fig. 4B; Fig. S12). Editing efficiencies were high (over 80% for impLbCas12a) within a 20 bp range upstream and downstream of the cleavage site. Compared to the wild-type LbCas12a, the efficiencies of impLbCas12a were higher or comparable at the tested canonical TTTC PAMs. The impact of the distance from the cleavage site on editing efficiency was consistent with wild-type LbCas12a targeting both strands. At approximately 30 bp from the cleavage site, the editing efficiencies dropped substantially for both variants (Fig. 4B). These results suggest that the editing window of impLbCas12a using the homology-integrated design is approximately 40 bp, centering around the cleavage site. This is on par with SpCas9 (27), suggesting that the editing range is mainly affected by the yeast homologous recombination process.

### High-throughput genetic variant engineering

To test the editing efficiencies of T-rich sequences, we attempted to edit the dihydrofolate reductase gene of the malaria parasite *Plasmodium falciparum* (*PfDHFR*), which has an extremely high AT content (74%). Dihydrofolate reductase is an essential oxidoreductase that converts dihydrofolate to tetrahydrofolate. Pyrimethamine (Pyr) inhibits this enzyme by competing with dihydrofolate for binding to the substrate binding pocket, thus killing the host cell. Several mutations in the binding pocket decrease the binding affinity toward Pyr, causing resistance. The *PfDHFR* gene can functionally complement the yeast endogenous dihydrofolate reductase gene. To construct a *PfDHFR* integrated yeast strain, we first introduced a frame-shift mutation to disrupt the yeast endogenous dihydrofolate reductase (*DFR1*) gene. We then integrated a GAP promoter-driven *PfDHFR* gene to the HOmothallic switching endonuclease (*HO*) safe harbor locus to derive a *PfDHFR* expression strain (BY4741-*DFR1*∆8bp-*HO::PfDHFR*). Several crRNAs were designed to edit the *PfDHFR* gene in this strain to obtain drug-resistant single amino acid mutants (Fig. S13). Efficient crRNAs were identified. *PfDHFR* C59R, S108N, and wild-type strains were subjected to spot assay in the presence of Pyr, which inhibits the growth of the wild-type *PfDHFR* strain, but not the *DFR1* strain BY4741 (Fig. S14). A significant increase in Pyr resistance of S108N mutants was observed, while C59R mutants did not develop resistance, consistent with a previous report (41).

**Fig 5:**
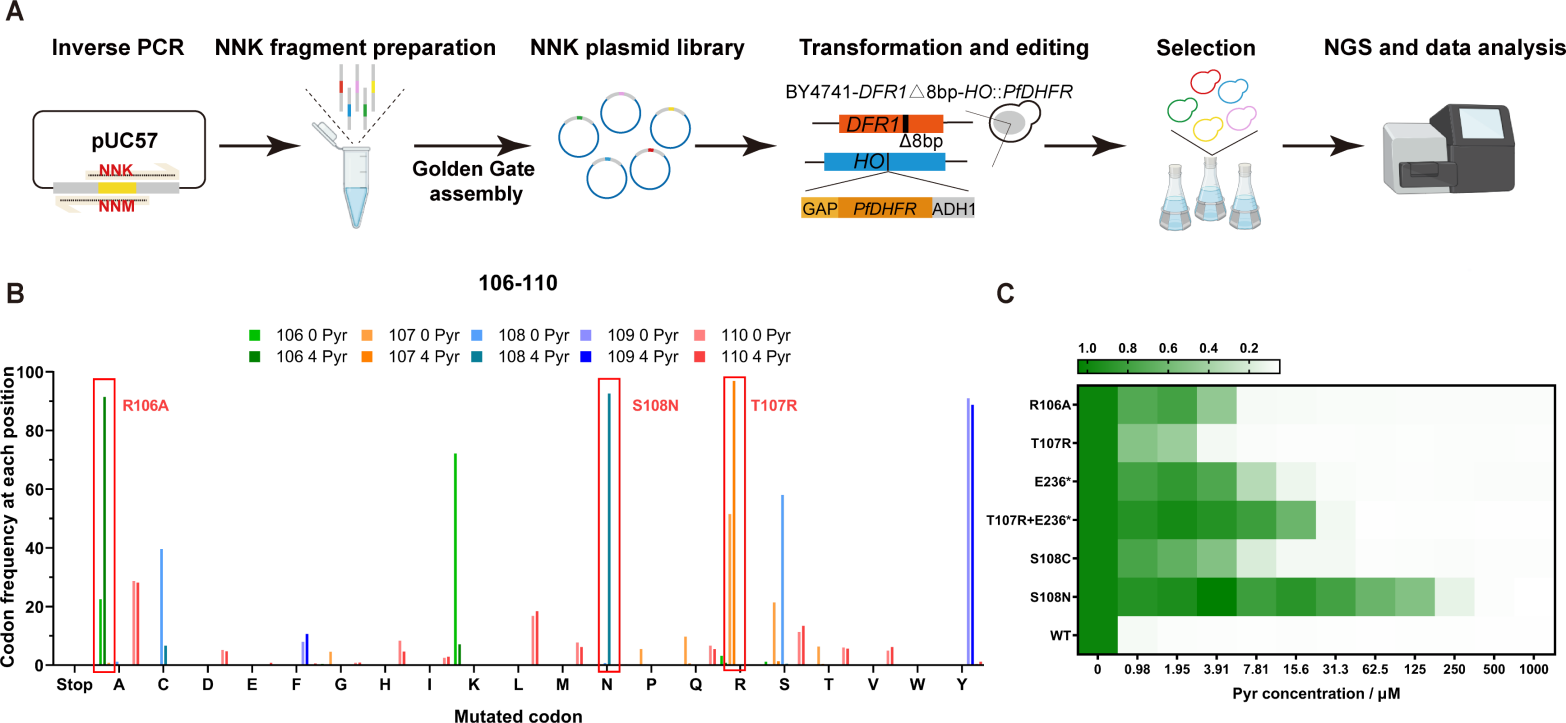
Saturation editing using the homology-integrated impLbCas12a system. (**A**) Workflow of saturation editing and drug selection of an NNK library scanning *PfDHFR* Arg106 to Glu110. (**B**) Enrichment of mutated codons after 4 µM pyrimethamine selection as assessed by next-generation sequencing. (**C**) Heatmap depicting the Pyr drug resistance of the reconstructed mutants. These strains all lack *DFR1*, and the labels refer to mutations in *PfDHFR*. The average optical density (OD) of three biological replicates for each sample is shown, with OD under no drug conditions normalized to 1.

To showcase high-throughput genetic variant engineering, we conducted saturation mutagenesis of five consecutive codons of *PfDHFR* using our system. An efficient crRNA (crPfDHFR-S108N-TATG, Fig. S15) was used to construct an NNK scanning library across codons encoding amino acid residues from Arg106 to Glu110, mutating each position to all 20 amino acids (Fig. 5A). The number of distinct plasmid constructs in the library is 160. The 15 bp editing region was designed to fall within the editing range of impLbCas12a as determined previously (Fig. 4B). We confirmed the presence of NNK at target locations in the plasmid library (Fig. S15). After yeast transformation and liquid cultivation, the yeast library was screened for mutations enhancing Pyr resistance. We first examined 24 colonies that appeared on drug-containing agar plates. All of them were mutants except for one wild-type strain. The mutants included 21 strains of S108C and 2 strains of T107R + E236* (where * indicates the stop codon). Alternatively, we enriched drug-resistant mutants in liquid cultivation containing the drug (Fig. 5A). The enriched populations as well as control populations without drug treatment were analyzed by next-generation sequencing (NGS). Results of untreated populations revealed an 89% coverage of all possible amino acid mutations (Table S5). Comparing the enriched populations with untreated populations revealed a decline in the proportions of unedited cells (59.1% in the untreated population versus 18.2% in the treated population). R106A, S108N, and T107R enriched substantially in the treated population, indicating that these mutants showed improved Pyr resistance (Fig. 5B). For the five mutations found and one double mutant, we designed crRNAs (Table S3) to reconstruct the strains and validated their increased Pyr resistance. S108N exhibited the highest resistance up to 125 µM Pyr in yeast extract peptone dextrose (YPD) media. The other mutants showed less but significant increase in resistance compared to the wild-type *PfDHFR* strain. The double mutant T107R + E236* exhibited a higher resistance than respective single mutants (Fig. 5C; Fig. S16). E236 is located in a flexible region near the C-terminus. We suspect that truncation at this position may have resulted in an increase in the enzyme expression level, leading to an increased drug resistance. A similar truncation, Q237*, was also found in an evolution experiment to be resistant in a previous study (42).

### Duplex genetic variant engineering

**Fig 6:**
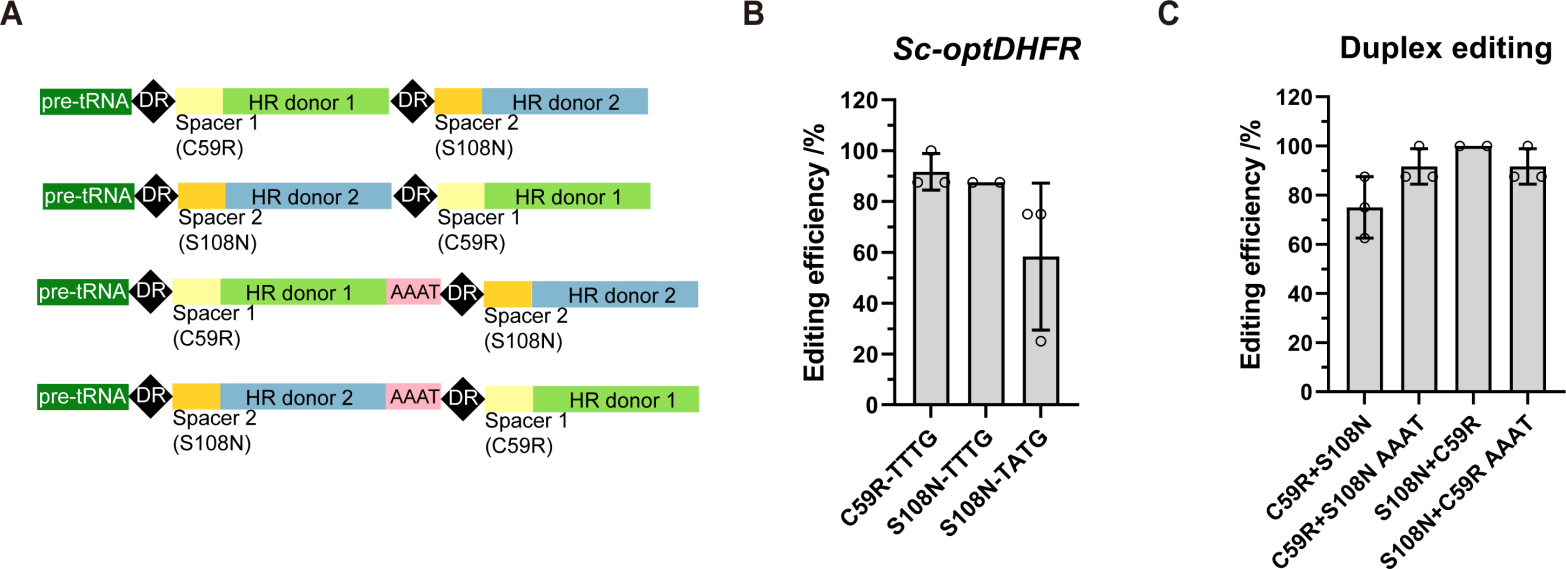
Duplex editing using the homology-integrated impLbCas12a system. (**A**) Design of duplex editing crRNA expression cassettes with alternating crRNA expression orders with or without the synSeparator (AAAT). (**B**) Editing efficiencies of individual crRNAs targeting *Sc-optDHFR*. (**C**) Duplex editing efficiencies of *Sc-optDHFR. Sc-optDHFR*, an integrated *S. cerevisiae* codon-optimized *DHFR* gene. *n* = 3 biological replicates. Error bars represent standard deviations. Some outliers were excluded.

To test the multiplex genetic variant engineering potential of our system, we attempted to introduce two codon replacements to the *PfDHFR* gene using a crRNA array. Two high-efficiency crRNAs, crPfDHFR-C59R-TTTG and crPfDHFR-S108N-TTTG (Fig. S13), were used to perform duplex editing. To explore crRNA positional effects, two duplex-editing plasmids with different crRNA orders were constructed (C59R + S108N and S108*N* + C59R, Fig. 6A). We also introduced synSeparators (AAAT) upstream of the second DR, which was reported to enhance crRNA array processing (43). To our surprise, none of the designs can edit effectively (Fig. S16). This is non-intuitive as each crRNA is efficient in single-locus editing. We suspect that, in comparison to the single crRNA construct, the dual-crRNA precursors may have formed complex secondary structures that prevented proper crRNA processing (Fig. S17). To avoid possible sequence-related failures, we additionally constructed a yeast strain with a *S. cerevisiae* codon-optimized copy of *PfDHFR* (*Sc-optDHFR*). We screened efficient and accurate crRNAs targeting C59 and S108 (crSc-optDHFR-C59R-TTTG and crSc-optDHFR-S108N-TTTG, Fig. 6B; Fig. S19). These two efficient crRNAs were used to construct duplex-editing plasmids with the same crRNA array designs (Fig. 6A). Sanger sequencing of dozens of yeast colonies from each design showed that the efficiencies of duplex editing were consistent with single-locus editing (Fig. 6C), demonstrating the functionality of the crRNA arrays. In addition, crRNA positional effects were negligible, and the synSeparator is also not required. These results were further confirmed by NGS to account for more colonies (Fig. S20). Thus, our system is capable of multiplex genetic variant engineering in yeast, given that efficient crRNAs can be screened.

## DISCUSSION

**Fig 7:**
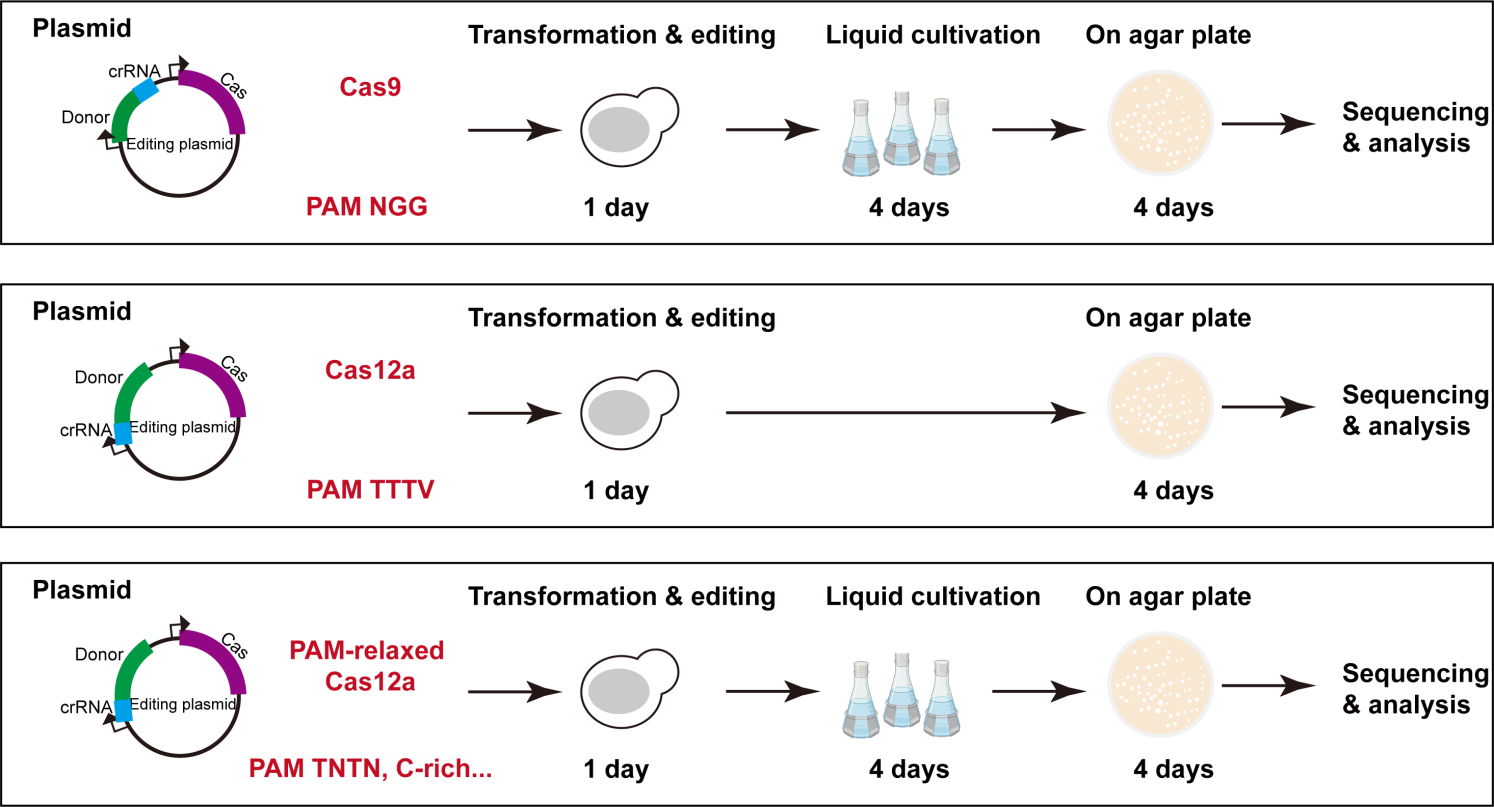
A comparison of Cas9 and Cas12a-based workflows for yeast genetic variant engineering. For Cas9, the donor is integrated 5′ of crRNA. It costs around 9 days to complete editing. For Cas12a, the donor is integrated 3′ of crRNA. When using the wild-type LbCas12a, the liquid cultivation can be omitted, which reduces the editing process by 4 days.

This study provides an integrated Cas12a-based tool for yeast genetic variant engineering. We integrated the homologous repair template at the 3′ end of the crRNA, enabling one-step assembly into a single plasmid encoding all necessary genetic parts, thereby simplifying the workflow for high-throughput library construction and chromosomal gene diversification (44). Compared with the Cas9-based method, this Cas12a system performs gene editing at distinct PAMs with comparable (when using impLbCas12a) or less time (when using wild-type LbCas12a) (Fig. 7). The crRNA expression design using a Pol II promoter with pre-tRNA in *S. cerevisiae* has been proven to achieve excellent single and multiplex guide RNA expression for metabolic pathway rewiring (45). In the present study, the Pol II promoter TEF1p was used to avoid potential premature transcriptional termination or weak expression of crRNAs. Additionally, fusing a pre-tRNA to the 5′ end of the crRNA enabled TEF1p-driven gene editing. In the future, other strong Pol II yeast promoters may be evaluated for their ability to further improve the editing efficiency of our system. Additionally, Pol III yeast promoters used for yeast gene editing are mainly restricted to the commonly used SNR52 and RPR1 promoters (9). The large repertoire of inducible and synthetic Pol II promoters may be utilized in our system to regulate crRNA expressions in a more controlled manner (46–49).

The characterized PAM compatibility of the impLbCas12a variant in this study is generally consistent with that in mammalian cells, with most of the reported PAMs being effectively recognized (Fig. 3B). However, it is important to note that for the same PAM, different spacer selections may lead to different editing efficiencies. LbCas12a-3Rv and LbCas12a-RR variants were consistent in recognizing a subset of reported PAMs (Fig. 2; Fig. S6). In preliminary tests of PrCas12a-3Rv and FnCas12a-EP16 in yeast, we did not observe any edited colonies (Supplementary Text; Fig. S7 and S8). This suggests that these variants need further optimization to work efficiently in yeast chromosomal environments. Recently, a resurrected ancestor of Cas12a (ReChb) derived from orthologs found in hydrobacterial phyla was shown to recognize NYYN, NRYN, and NYRN PAMs in eukaryotic cells (50). Such PAM-relaxed Cas12a variants may be tested in our system to further expand the PAM recognition space.

Saturation mutagenesis and screening of *PfDHFR* identified four previously unreported Pyr-resistant mutations—R106A, T107R, S108C, and E236*—in addition to the well-documented S108N mutation. While E236* (GAA to TAA) should be a random mutation being enriched, all other four mutations within the target window involve the substitution of two or three nucleotides of the wild-type codon (Fig. S21). This, together with the lower resistance of the newly found mutations, might be the reason why they were not observed in previous studies using T7 RNA polymerase-guided base editing (42) or random *in vivo* mutagenesis (41), where the frequency of concurrent two or three base changes is low and the mutations are not trackable through high-throughput sequencing due to the low mutation rates (on the orders of 10^−4^ substitutions per base). This is particularly evident for R (with codons CGN and AGR) to A (with codons GCN) mutation, which cannot be achieved by mutating a single nucleotide. Thus, our system is more advantageous in examining a greater diversity in user-defined regions by framing the target range through the selection of proper crRNAs and the writing of synthetic mutations in the donors. Such a targeted strategy should be advantageous in reducing screening burdens if relevant structural information is available (51).

Finally, we successfully achieved duplex genetic variant engineering using a single plasmid expressing a homology-integrated crRNA array (Fig. 6). This could enable future research endeavors aiming to dissect the functional effects of combinatorial genetic variants across user-selected genomic regions, genes, or protein domains. Our system can also be extended to encompass further multiplexing, given that four crRNAs were successfully expressed in previous yeast studies (22), and even more crRNAs (25) have been successfully expressed in a single array in mammalian cells (52). Pol II promoters enable the expression of over 4,000 nt sequences (53, 54), sufficient for expressing multiple guide + donor combinations.

We note that not all crRNAs are of high efficiency in this study (Fig. 6B; Fig. S13). In addition, inefficient crRNAs were not rescued by introducing further mutations into impLbCas12a (Supplementary Text; Fig. S22 to S24). Recent developments in crRNA efficiency prediction algorithms customized particularly for yeast may improve the accuracy of crRNA selection (55, 56). Alternatively, crRNA engineering may be a viable option for rescuing inefficient spacers. For example, researchers recently introduced Z bases into crRNA, replacing A:T with Z:T base pairs that contain three hydrogen bonds (57), which significantly improved the editing efficiency of the CRISPR/Cas12a system.

Our system may be extended to other hosts, such as industrial *S. cerevisiae* strains and non-conventional yeasts. In industrial polyploid *S. cerevisiae* strains, simultaneous editing of multiple alleles or genes is readily achievable with Cas9 (58). Pol II promoters have been demonstrated to effectively express crRNAs for gene editing in non-conventional yeasts (23, 24). In addition, HDR efficiencies can be boosted in non-conventional yeasts using multiple strategies. For example, previous studies have demonstrated that perturbing DNA repair pathways, including knocking out the *Ku70-Ku80* heterodimer, which mediates NHEJ (59, 60), or overexpressing HR-related genes such as *RAD52* (61, 62) increased HDR efficiencies in non-conventional yeasts. The lowered indel nuclease system enabling accurate repair platform (63) enhanced the HDR efficiencies in three non-conventional yeast species by circumventing the NHEJ pathway through fusing a cell cycle protein to Cas9. The above strategies may be combined with our system to enable Cas12a-assisted genetic variant engineering in yeasts other than *S. cerevisiae*.

## MATERIALS AND METHODS

### Strains and media

*E. coli* Trelief 5α (Cat. #TSC-C01; Tsingke, Hangzhou, China), TOP10 (Cat. #TSC-C12, Tsingke), and DH5α Electro-Cells (Cat. #9027; Takara Biotechnology, Dalian, China) were used for plasmid construction. Luria-Bertani (LB) media (10  g/L tryptone, 10  g/L NaCl, and 5  g/L yeast extract) with 100 µg/mL ampicillin was used for plasmid amplification. All *E. coli* strains were incubated at 37°C, 200 rpm. The *S. cerevisiae* strain BY4741 (MATa *his3Δ0 leu2Δ0 met15Δ0 ura3Δ0*) was used to derive BY4741-*DFR1*∆8bp, BY4741-*DFR1*∆8bp-HO::*PfDHFR*, and BY4741-*DFR1*∆::*Sc-optDHFR*. YPD medium (20  g/L tryptone, 20  g/L glucose, and 10  g/L yeast extract) and YPAD medium (YPD medium supplemented with 0.1 g/L 6-aminopurine hemisulfate) were used for yeast culture. The synthetic complete minus uracil (SC-Ura) medium was used for yeast transformant selection and growth. YPD medium with indicated pyrimethamine (Cat. #P141438; Aladdin, Shanghai, China) concentration was used for drug resistance screening and testing. All yeast strains were incubated at 30°C at 250 rpm.

### Plasmid construction

The crRNA consists of a DR associated with Cas12a of different species (Table S1) and a 23 nt spacer. The pre-tRNA^Gly^ (77 bp) was from *S. cerevisiae*. The repair donor was added to the 3′ end of crRNA. The repair donor contains two 50 bp homology arms flanking the mutated region. TEF1p and CYC1t were used as the promoter and the terminator for crRNA expression. LbCas12a variants were constructed by site-directed mutagenesis of the wild-type template (pH5-NLS-LbCpf1-NLS, a kind gift from Dr. Jiazhang Lian, Zhejiang University). FnCas12a-EP16 was amplified from pET28TEV-FnCpf1-EP16 (a kind gift from Dr. Yunzi Luo, Tianjin University). PrCas12a-3Rv and ScFnCas12a-EP16 fragments (FnCas12a-EP16 with *S. cerevisiae* codon optimization) and all crRNAs (with repair donor) used in this study were commercially synthesized (SynbioB, Tianjin, China). LbCas12a-3Rv gene fragment was derived by referencing enAsCas12a mutations through sequence alignment. The Cas12a expression cassette and pre-tRNA fragments were first ligated into pCRCT (Addgene plasmid #60621) by Gibson Assembly (Cat. #E2611; NEB, Hangzhou, China) to generate a series of plasmids with Cas12a variants (pCRCT-impLbCas12a, pCRCT-LbCas12a-RR, pCRCT-LbCas12a-3Rv, pCRCT-LbCas12a-3Rv, pCRCT-PrCas12a-3Rv, and pCRCT-ScFnCas12a-EP16). The crRNA receiver part (with *Bsa*I restriction sites) and the DR sequence were commercially synthesized (GenScript, Nanjing, China) and subsequently cloned into the above pCRCT-Cas12a plasmids to generate pCRCT-Cas12a-receiver plasmids. Each crRNA plus donor was assembled into the respective receiver plasmid using Golden Gate assembly (Cat. #R3733S and Cat. #M0202A, NEB). For plasmids expressing dual crRNAs, each crRNA plus donor fragment was amplified by primers containing *Bsa*I restriction sites, purified, and assembled with receiver plasmid using Golden Gate assembly. Linear donors were PCR amplified using corresponding donor-integrated plasmids as templates. Plasmids without integrated donors were amplified using reverse-PCR and circularized to remove the donor sequence.

### Yeast transformation

Yeast transformation was conducted using the LiAc/ssDNA/PEG method. For single plasmid transformation, 1 µg plasmid was used per transformation of approximately 5 optical densities (ODs) of yeast cells. For plasmid library transformation, 24 µg of the plasmid library was used per transformation of 25 ODs of yeast cells. Ten transformations were performed to maximize the library coverage. Transformed yeast cells were grown in 10 mL SC-Ura in a 50 mL flask at 250 rpm at 30°C for 4 or 5 days before pooling for storage or drug screening. For plasmid library transformation, three transformations were diluted and plated to estimate transformation efficiency. The total number of transformants was estimated to be 1.6 × 10^4^, representing a 100-fold coverage of the library size.

### Yeast mutant genotyping

Genomic DNA extracts of selected yeast mutants or pools were prepared using the MightyPrep Reagent for DNA (Cat. #9182; Takara Biotechnology, Hangzhou, China). Target gene fragments were PCR amplified using corresponding primers (Table S2) and the genomic DNA extract as template, purified, and sequenced using either Sanger sequencing or next-generation sequencing to determine the genotypes.

### Construction of PAM-replaced strains and PAM specificity test

The PAM (TATA) of a high-efficiency crRNA (crADE2-TATA, Table S3) near the protospacer of crADE2-PAMchange (Table S3) was selected for replacement to TNTN (TATG/TATT/TCTG/TCTT/TGTC/TGTG/TGTT/TTTT). crADE2-PAM change plus donors with TNTN PAMs and a stop codon were constructed by commercial DNA synthesis and site-directed mutagenesis on the pUC57 backbone and subcloned into respective pCRCT-Cas12a backbones. After transformation and editing, red colonies were picked to confirm the genotypes by Sanger sequencing. The editing plasmid was cured by growing an edited colony in YPD supplemented with 2 mg/mL 5′-FOA for 2 days. For specificity testing, a plasmid expressing crADE2-TATA with a sequence-correcting donor (PAMtest, Table S3) was transformed into these plasmid-cured strains to test editing efficiencies at the eight TNTN PAMs.

### Construction of the *DHFR* integration strains

The SpiG variant (14) was used to integrate *DHFR* into BY4741 genome. gRNA-*DFR1*∆8bp (Table S1) was designed to delete eight nucleotides (GGAGGTCT) within the endogenous *DFR1* gene and cloned into the pCRCT-gRNA scaffold (14). BY4741-*DFR1*∆8bp was obtained after transformation, editing, and genotyping. gRNA-HO (Table S1) was designed to target the *HO* integration locus and cloned into the pCRCT-gRNA scaffold. This plasmid was co-transformed with a commercially synthesized linear dsDNA fragment, GAPp-*PfDHFR*-ADH1t (Table S1), into BY4741-*DFR1*∆8bp to derive BY4741-*DFR1*∆8bp-*HO::PfDHFR*. Similarly, an *S. cerevisiae* codon-optimized *DHFR* fragment (*Sc-optDHFR*) with 50 bp flanking homology arms (Table S1) was co-transformed with the plasmid expressing SpiG and gRNA-*DFR1* to derive BY4741-*DFR1*∆::*Sc-optDHFR*.

### Construction of the NNK plasmid library

For each target codon from amino acid positions 106–110, a pair of degenerate primers (NNK at the target codon position) was synthesized and used to amplify the crRNA (crPfDHFR-S108N-TATG, Table S3) in a pUC57 plasmid backbone using KOD One PCR Master Mix (Cat#. KMM-101; TOYOBO Shanghai, Japan). The PCR cycling conditions were 98°C for 5 min (98°C for 10 s, 58°C for 5 s, and 68°C for 20 s) × 24 cycles, 68°C for 2 min, and held at 12°C. After DpnI (Cat. #R0176S, NEB) digestion and purification, each PCR product was transformed into DH5α Electro-Cells and spread on one 24 × 24 cm LB agar plate with 100 µg/mL ampicillin. After 14 h of incubation at 37°C, all colonies from the five plates were separately collected for plasmid extraction. The crRNA fragments were amplified by crRNA-amplify-F1/R1 (Table S2) using 10 ng of extracted plasmid libraries as templates. The PCR cycling conditions were 98°C for 5 min (98°C for 10 s, 58°C for 5 s, and 68°C for 5 s) × 24 cycles, 68°C for 2 min, and held at 12°C. Purified crRNA fragments from all five PCR reactions were mixed in equal molar ratio. The mixture (15 ng) was used to assemble with 50 ng of pCRCT-impLbCas12a-receiver via Golden Gate assembly. Golden Gate products were purified and transformed into Electro-Cells and plated onto LB agar plates. Five such transformations were conducted to increase the library coverage. A library coverage of approximately 1,000-fold was estimated by colony counting. The final plasmid library was obtained by maxiprepping the scraped colonies.

### Yeast library selection

For selection on plate, serial dilutions of the outgrown NNK mutant library were spread on 90 mm YPD plates supplemented with 10 µM Pyr. After 5–6 days of incubation at 30°C, colonies were randomly selected for genotyping by Sanger sequencing. For selection in liquid media, the glycerol stock of the NNK mutant library was first recovered in YPD overnight. The next morning, 2 ODs of recovered cultures were inoculated into 30 mL YPD medium with and without 4 µM Pyr, for three biological replicates. After 2 days of growth, three replicates at each condition were mixed at equal OD and used to amplify genomic DNA.

### Next-generation sequencing

The 258 bp fragment of *PfDHFR* was amplified with primers 106-110NNK-pool-TF1/TR1 (Table S2) using KOD Plus Neo (Cat. #KOD-401, TOYOBO Shanghai) and 10 ng yeast genomic DNA as template. The PCR procedures were 98°C for 5 min (98°C for 10 s, 58°C for 30 s, and 68°C for 6 s) × 20 cycles, 68°C for 2 min, and held at 12°C. The PCR products were gel purified (Cat. #K0702; Thermo Fisher Scientific, Hangzhou, China). Samples were paired-end sequenced for 150 cycles on Illumina Novaseq (Azenta, Suzhou, China). For comparing the editing efficiencies of impLbCas12a and hyper + impLbCas12a using NGS, three uniquely barcoded pairs of primers (Table S2) were used to amplify a 200 bp *PfDHFR* fragment covering the D54N edit for each biological replicate. Equal amounts of the three PCR products were mixed as NGS samples. Samples were paired-end sequenced for 150 cycles on Illumina Novaseq.

### Next-generation sequencing data analysis

Raw NGS data were generated using Illumina bcl2fastq. Paired-end reads were merged and adapters were trimmed using Merging paired-end Illumina reads (SeqPrep) in Galaxy (https://usegalaxy.eu) with default settings. Clean reads were subsequently analyzed using custom codes (https://zenodo.org/records/14676182). Briefly, merged reads were first filtered by the presence of primer sequences and subsequently oriented according to the sense strand. For saturation mutagenesis, all types of non-wild-type codons within the 106–110 region were counted. Subsequently, the counts were converted to percentages of total counts at each position. For comparing the editing efficiencies of impLbCas12a and hyper+impLbCas12a, the read numbers of the wild-type and edited sequences were counted, and the percentage of edited sequences was calculated to determine the editing efficiency.

### Pyrimethamine resistance test

A streaked colony of each strain was inoculated into 2 mL YPD overnight. Minimum inhibitory concentration assay was used to measure drug resistance levels. Briefly, 50 µL of the diluted overnight cultures was added to each well containing 100 µL YPD supplemented with increasing concentrations of Pyr (from 0 to 1,000 µM final concentrations) in a 96-well plate, with an initial OD of 0.04. The OD_600_ of three replicates was measured at 0 and 48 h of incubation at 30°C at 250 rpm using Agilent BioTak Synergy H1 (Agilent, Beijing, China). After averaging, the 0 h data were extracted from the 48 h data. All data were normalized to the OD numbers under 0 Pyr concentration for each strain.

## Data Availability

Raw sequencing reads were deposited at the National Center for Biotechnology Information Sequence Read Archive with accession number PRJNA1211711.

## SUPPLEMENTAL MATERIAL

Supplemental material aem.01618-25-s0001.pdf Supplemental text, Fig. S1 to S24, and Tables S4 and S5.

Table S1 aem.01618-25-s0002.docx List of DNA and protein sequences.

Table S2 aem.01618-25-s0003.xlsx List of primers.

Table S3 aem.01618-25-s0004.xlsx List of crRNA with donor.

ASM does not own the copyrights to Supplemental Material that may be linked to, or accessed through, an article. The authors have granted ASM a non-exclusive, world-wide license to publish the Supplemental Material files. Please contact the corresponding author directly for reuse.
