# Supplementary material for: Benchmarking the PAM compatibility of Cas12a variants for high-throughput yeast genetic variant engineering
Source: Appl Environ Microbiol. 2025 Nov 25;91(12):e01618-25. doi: 10.1128/aem.01618-25 (PMC12724276; doi:10.1128/aem.01618-25)
Supplement: Supplemental material — Supplemental text, Fig. S1 to S24, and Tables S4 and S5. [file aem.01618-25-s0001.pdf]

SUPPLEMENTARY INFORMATION

Benchmarking the PAM compatibility of Cas12a variants for high-throughput yeast genetic variant engineering

Weiyu Xie,[1,2,3] Zhenkun Cai,[1,2,3], and Zehua Bao[1,2,3,4]*

[1]Key Laboratory of Biomass Chemical Engineering of Ministry of Education, College of Chemical and Biological Engineering, Zhejiang University, Hangzhou, 310058, Zhejiang, China.

[2]Zhejiang Key Laboratory of Intelligent Manufacturing for Functional Chemicals, ZJU-Hangzhou Global Scientific and Technological Innovation Center, Zhejiang University, Hangzhou, 311215, Zhejiang, China.

[3]Institute of Bioengineering, College of Chemical and Biological Engineering, Zhejiang University, Hangzhou, 310058, Zhejiang, China.

[4]Zhejiang Key Laboratory of Smart Biomaterials, College of Chemical and Biological Engineering, Zhejiang University, Hangzhou, 310058, Zhejiang, China.

*Correspondence should be addressed to Z.B. (zbao@zju.edu.cn).

SUPPLEMENTARY TEXT

Characterizing the PAM compatibility of FnCas12a-EP16 and PrCas12a-3Rv in *S. cerevisiae*

Besides LbCas12a variants, we also tested FnCas12a-EP16 (1), an in vitro validated PAM-relaxed variant of FnCas12a (**Table S4**). crRNAs were designed targeting six PAMs (NNTG, NNTA, NTCA, TCTC, NNTC, and GTTG). However, the editing efficiencies were negligible (**Fig. S7**). Subsequently, the direct repeat sequence of LbCas12a in the original plasmid was replaced with the DR of FnCas12a, and FnCas12a-EP16 was replaced by ScFnCas12a-EP16 (with *S. cerevisiae* codon optimization). However, we still did not observe edited colonies. Additionally, we observed that the transformation efficiencies associated with FnCas12a-EP16 were significantly lower than with LbCas12a variants and the growth rate of transformants was also substantially reduced, a phenomenon also seen in a previous study (2).

PrCas12a-3Rv was reported to recognize VTTV, TTCV, and TRTV non-canonical PAMs other than the canonical TTTV PAMs (3). We synthesized a *S. cerevisiae* codon-optimized PrCas12a-3Rv gene and designed crRNAs for testing at NTTG PAMs. However, the results showed no editing other than at TTTG (**Fig. S8**). After replacing the DR of LbCas12a with the DR of PrCas12a, we still did not observe edited colonies.

Preliminary testing of hyper+impLbCas12a

During the testing of crRNAs targeting the *PfDHFR* gene, we observed that some crRNAs

exhibited nearly zero editing efficiency (**Fig. S10**). Therefore, we attempted to introduce reported mutations into the PAM-relaxed impLbCas12a variant to improve nuclease activity by enhancing base interactions. Referring research of HyperCas12a (4), impLbCas12a+E292R and hyper+impLbCas12a were constructed. Inefficient crRNAs targeting *PfDHFR* were selected and assembled into the pCRCT-Cas12a receiver plasmids respectively (with impLbCas12a, impLbCas12a+E292R, and hyper+impLbCas12a variants) to obtain the editing plasmids. After yeast transformation, transformants were randomly selected for sanger sequencing to calculate editing efficiencies. The results indicated that these mutations (D235R/E292R/D350R) did not improve the efficiency on top of imp mutations with the tested crRNAs (**Fig. S22**). Four additional low-efficiency crRNAs targeting the *Sc-optDHFR* gene were selected to construct editing plasmids. Pools of yeast transformants were analyzed by next generation sequencing. The NGS data revealed that the hyper+impLbCas12a variant achieved an improved efficiency with crSc-optDHFR-D54N-TTTG, which showed significantly low efficiencies when using impLbCas12a (**Fig. S23**). Significant improvements were also observed for non-canonical PAMs within *ADE2* (**Fig. S24**). This suggests that hyperCas12a mutations improved editing efficiency of impLbCas12a.

SUPPLEMENTARY FIGURES

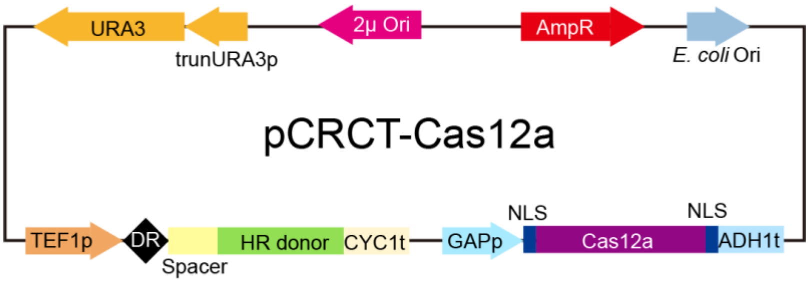

Supplementary Figure 1. Map of pCRCT-Cas12a plasmid used in the study. TEF1p, TEF1 promoter; DR, direct repeat; CYC1t, CYC1 terminator; GAPp, GAP promoter; NLS, nuclear localization sequence; ADH1t, ADH1 terminator; Ori, origin; AmpR, ampicillin resistance marker; trunURA3p, a truncated URA3 promoter.

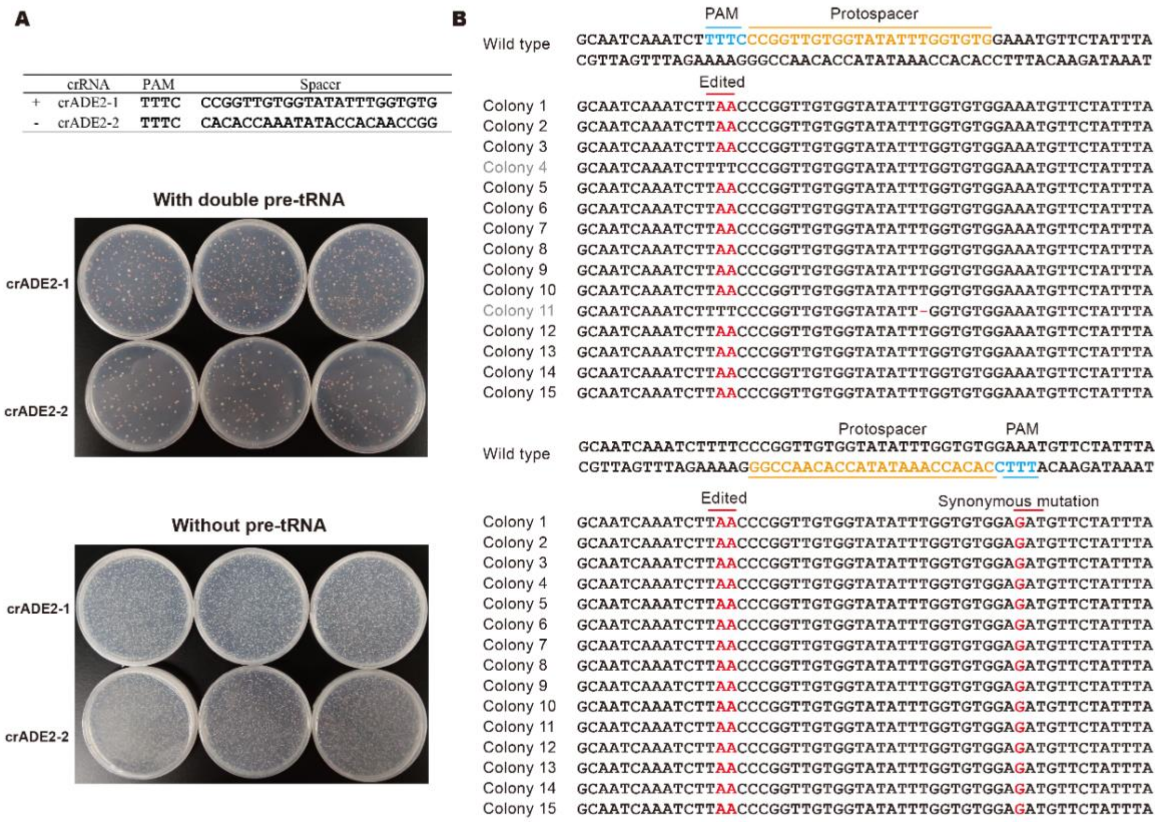

Supplementary Figure 2. ***ADE2*** **editing results related to figure 1c.** (**A**) Pictures showing edited (red) yeast colonies. (**B**) Sequencing results of fifteen randomly picked red colonies for each crRNA with double pre-tRNA.

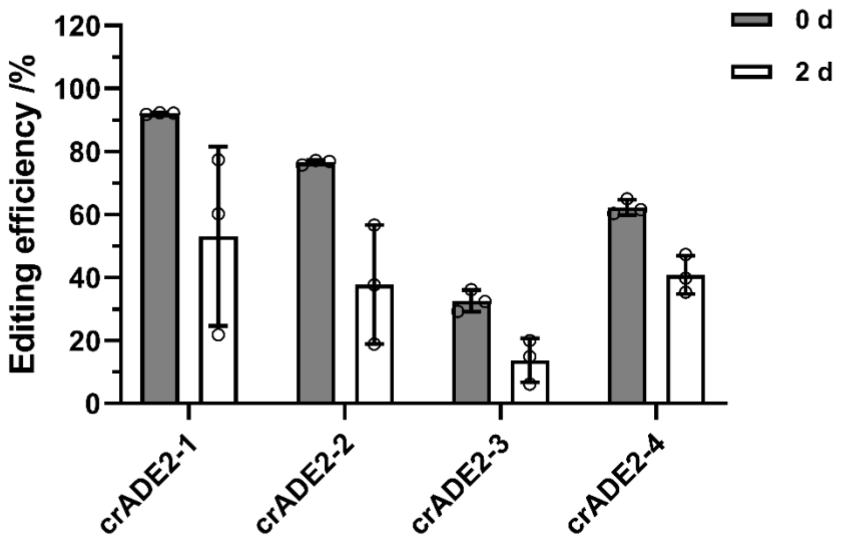

Supplementary Figure 3. Four crRNAs targeting *ADE2* were used to assess the impact of incubation time after transformation on the editing efficiency of wild-type LbCas12a. 0 d, zero day after transformation; 2 d, cells were plated after two days of liquid culture of the transformants. n = 3 biological replicates. Error bars represent standard deviations.

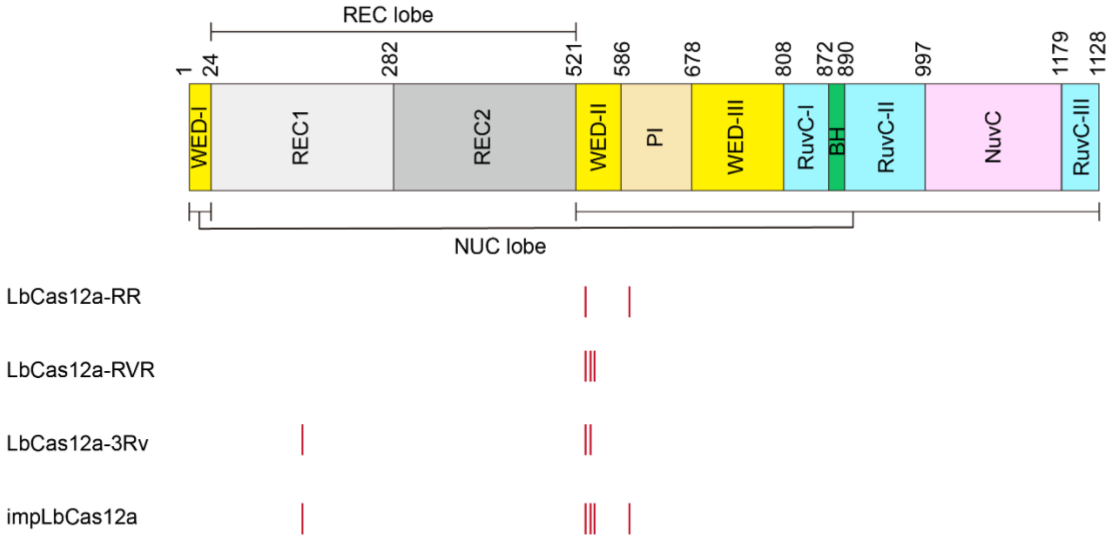

Supplementary Figure 4. The domain architecture of LbCas12a and location of mutations in different variants. REC, recognition; NUC, nuclease; WED, wedge; PI, pam interaction domain; BH, bridge helix.

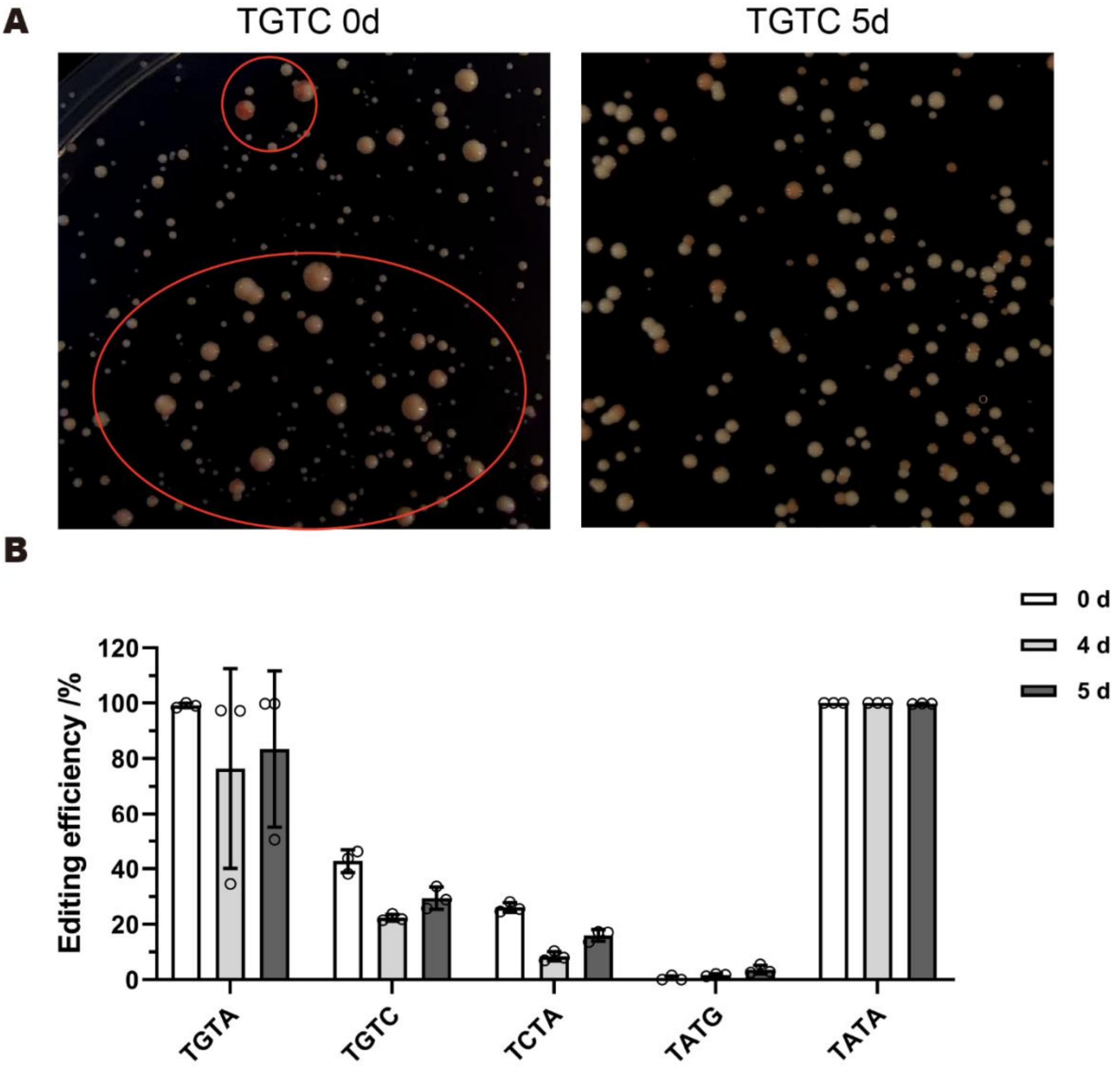

Supplementary Figure 5. ***ADE2*** **editing using the homology-integrated impLbCas12a system.** (**A**) Picture of yeast colonies on SC-Ura plate without or with liquid incubation. Representative chimeric colonies are circled. TGTC, crADE2-TGTC (Supplementary Table S3). 0d, zero days of incubation after transformation; 5d, five days of incubation after transformation. (**B**) The impact of liquid incubation time on editing efficiency at non-canonical PAMs. 0 d, zero days of incubation; 4 d, four days of incubation; 5 d, five days of incubation. The editing efficiencies with 0 d incubation include red and chimeric colonies. n = 3 biological replicates. Error bars represent standard deviations.

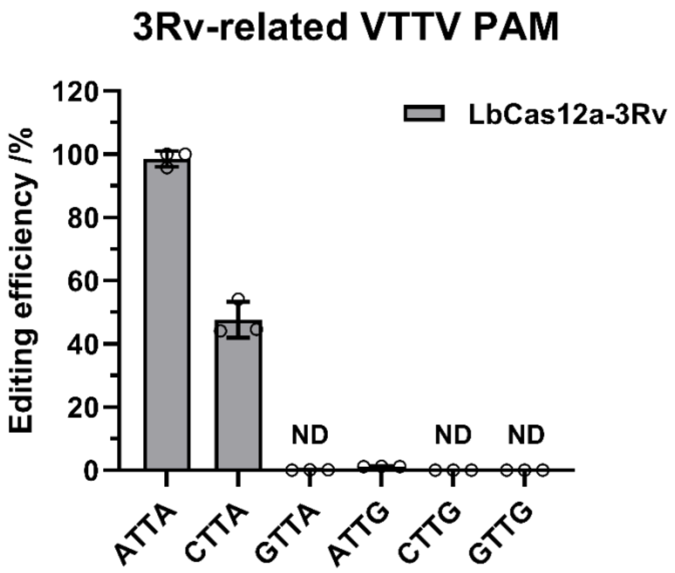

Supplementary Figure 6. The *ADE2* editing efficiencies of LbCas12a-3Rv at VTTV PAMs.

ND, not detected. n = 3 biological replicates. Error bars represent standard deviations.

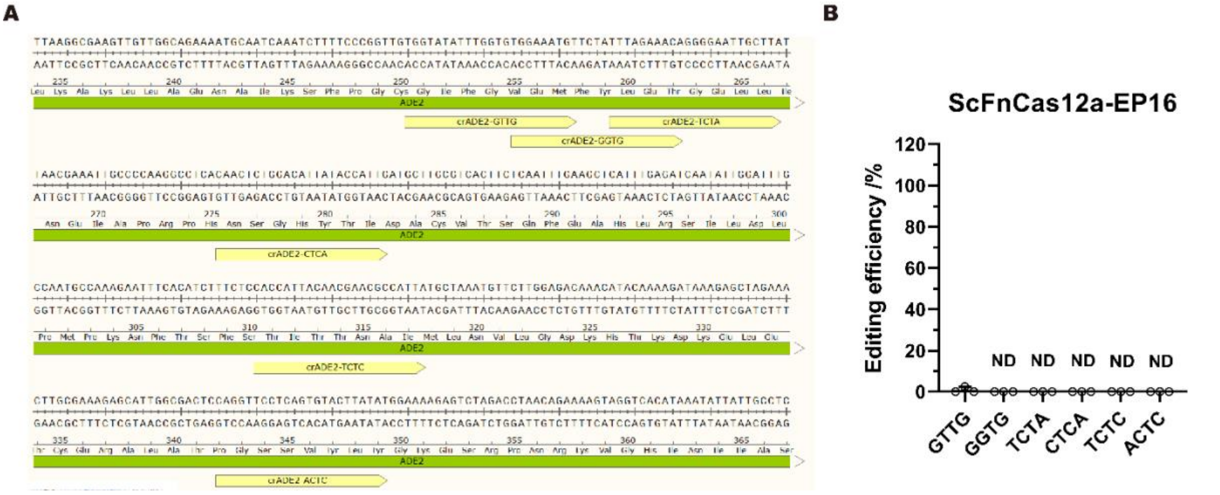

Supplementary Figure 7. (**A**) The spacer sequences of crRNAs used in the testing of ScFnCas12a-EP16. (**B**) The *ADE2* editing efficiencies of ScFnCas12a-EP16 at indicated PAMs. ND, not detected. n = 3 biological replicates. Error bars represent standard deviations.

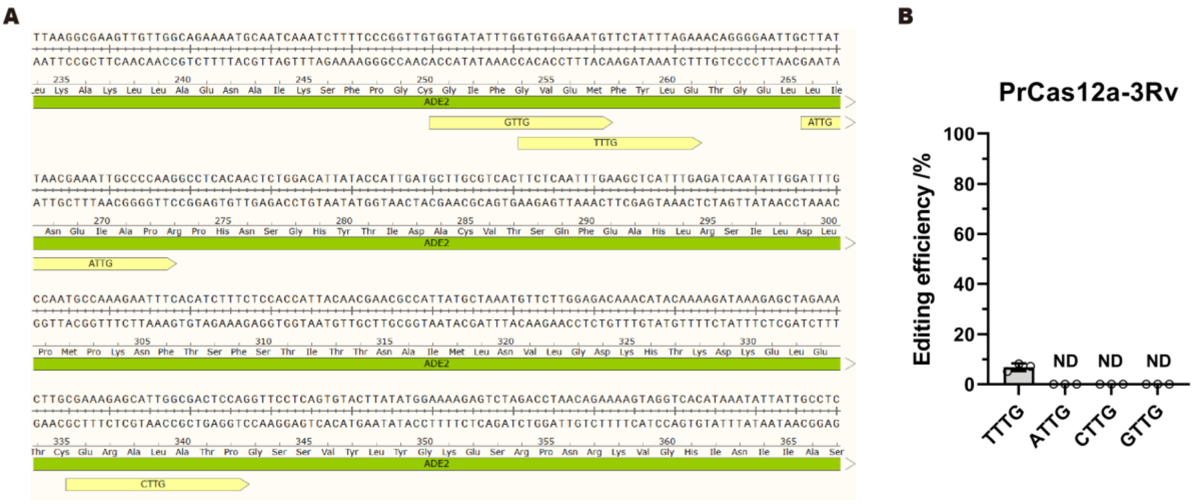

Supplementary Figure 8. (**A**) The spacer sequences of crRNAs used in the testing of PrCas12a-3Rv. (**B**) The *ADE2* editing efficiencies of PrCas12a-3Rv at indicated PAMs. ND, not detected. n = 3 biological replicates. Error bars represent standard deviations.

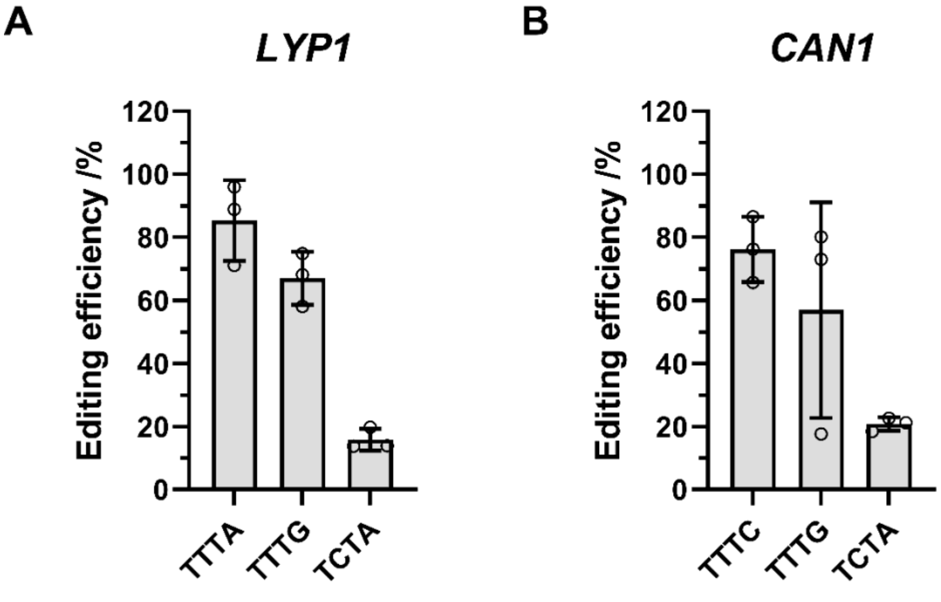

Supplementary Figure 9. (A) *LYP1* editing efficiencies of impLbCas12a. n = 3 biological replicates. **(B)** *CAN1* editing efficiencies of impLbCas12a. n = 3 biological replicates. Error bars represent standard deviations.

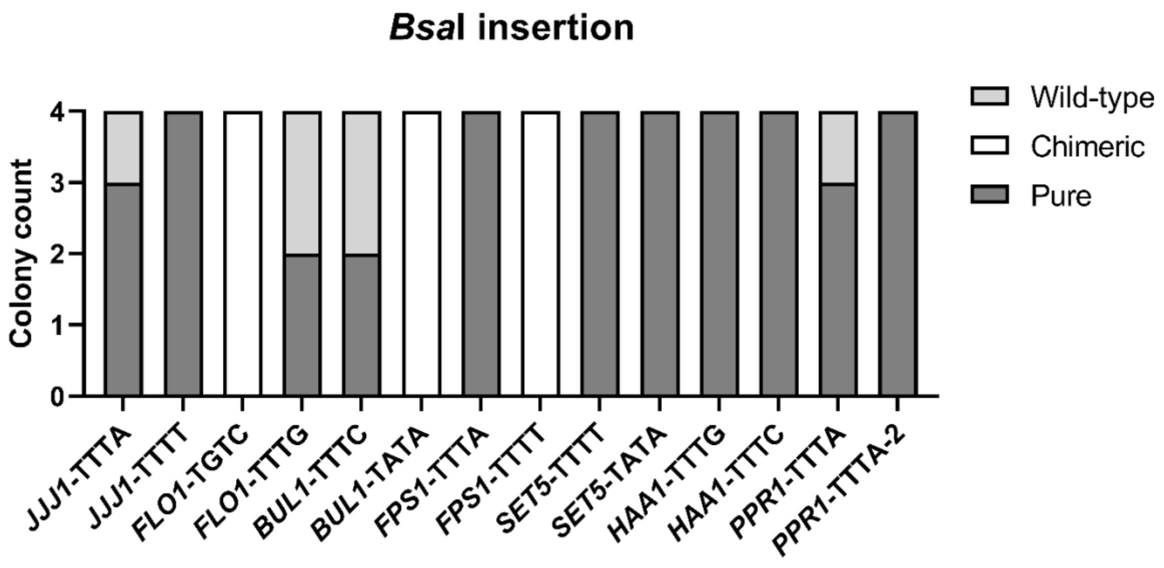

Supplementary Figure 10. The editing efficiency and purity of impLbCas12a in integrating a pair of *Bsa*I restriction sites into the 5'-UTR of endogenous yeast genes**.** Colonies in each group were randomly picked and analyzed by Sanger sequencing.

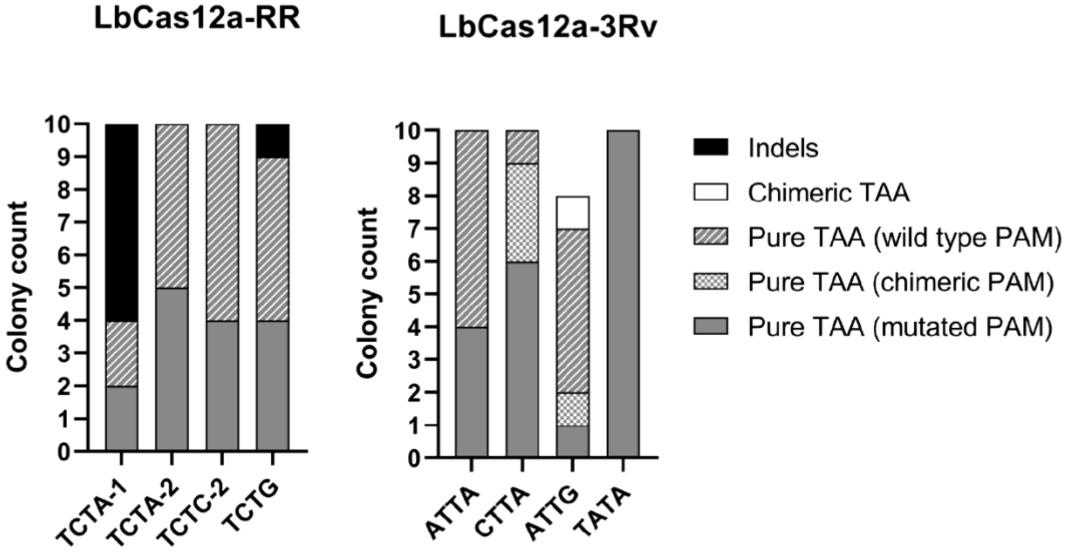

Supplementary Figure 11. Editing purity of LbCas12a-RR and LbCas12a-3Rv. Red colonies in each group were randomly picked and analyzed by Sanger sequencing.

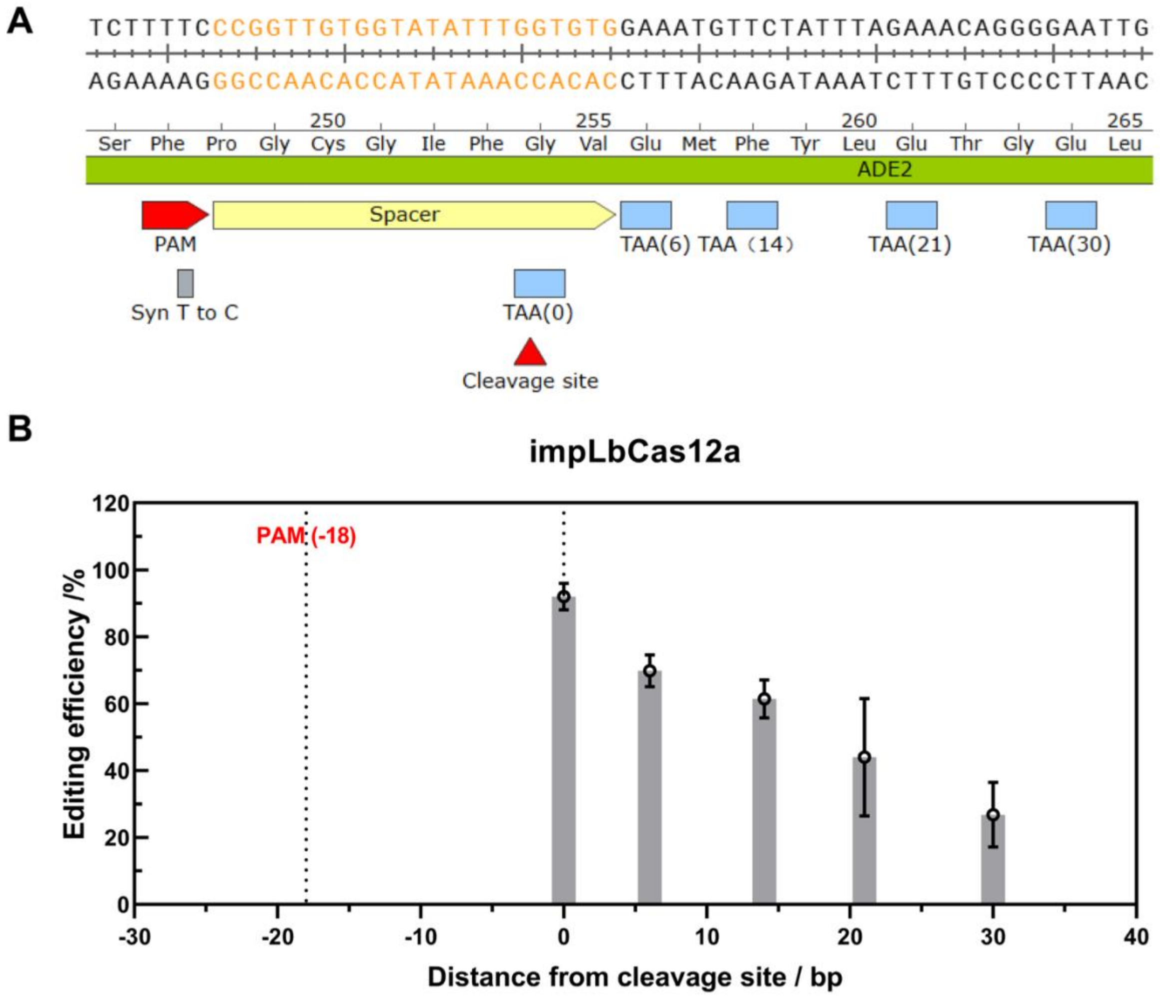

Supplementary Figure 12. Editing range of impLbCas12a downstream of the + strand crRNA in Figure 4A. (**A**) The design of donors with varying distances between the edit and the cleavage site. (**B**) Editing efficiencies at different distances. n = 3 biological replicates. Error bars represent standard deviations.

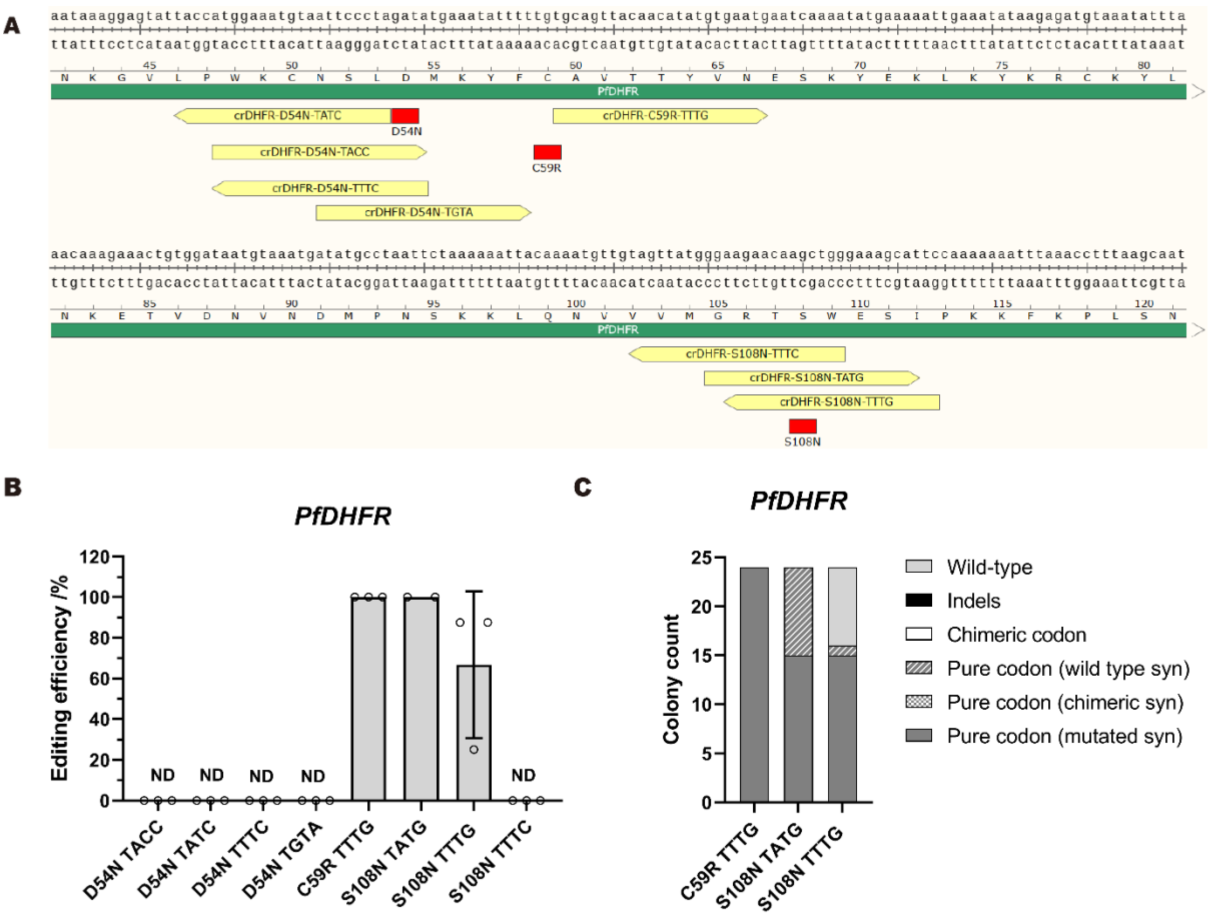

Supplementary Figure 13. Genomic editing of *PfDHFR*. (**A**) The spacer sequences of crRNAs used to target *PfDHFR*. (**B**) Editing efficiencies assessed by Sanger sequencing of randomly picked colonies. ND, not detected. n = 3 biological replicates. Error bars represent standard deviations. (**C**) Editing accuracy of the three efficient crRNAs. syn, the designed synonymous mutations near the PAM.

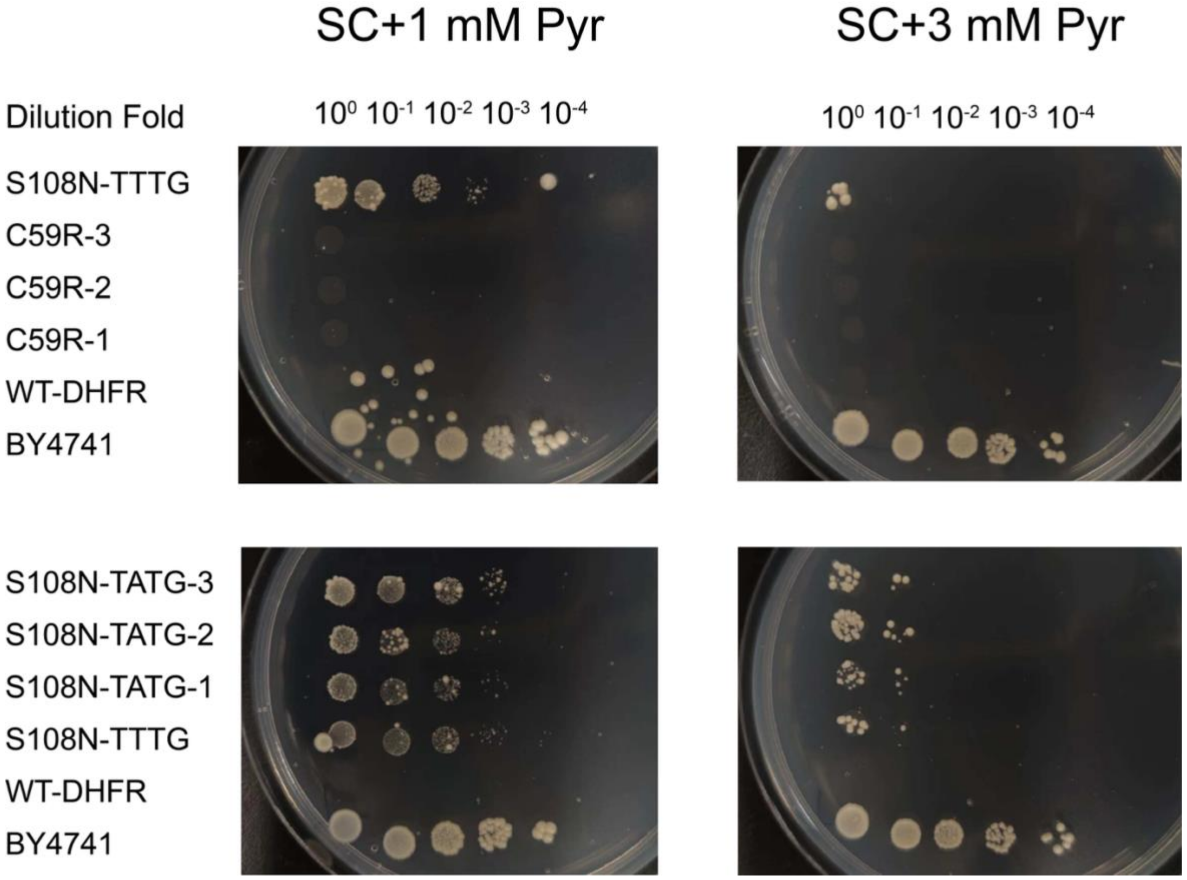

Supplementary Figure 14. Spot assay of BY4741, WT-*DHFR* (BY4741-*DFR1*Δ8bp-*HO*::*PfDHFR*), *PfDHFR* C59R, and *PfDHFR* S108N yeast strains with different Pyr concentrations. Pictures were taken three days after spotting. The OD of the undiluted spot is normalized to 0.5 with a spotted volume of 5 µl. C59R-1, C59R-2, and C59R-3 represent three independently edited colonies from Supplementary Figure 13B (C59R TTTG). S108N-TTTG, S108N-TATG-1, S108N-TATG-2, and S108N-TATG-3 represent independently edited colonies from Supplementary Figure 10B (S108N TTTG and S108N TATG).

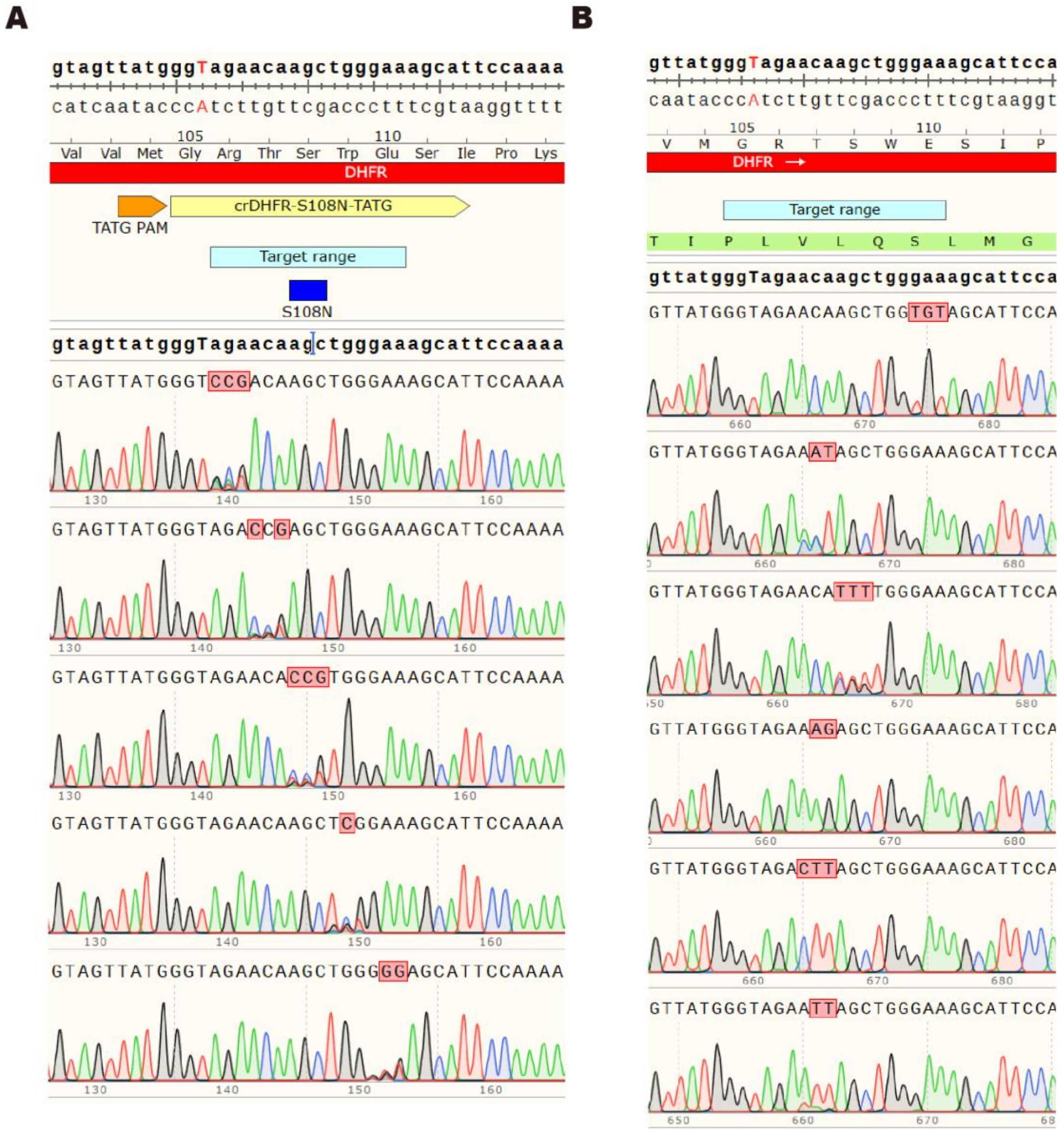

Supplementary Figure 15. (**A**) The Sanger sequencing result of five pUC57 plasmid libraries. (**B**) The sequencing results of six colonies randomly picked from the final plasmid library. The nucleotide in red indicates the synonymous mutation to prevent Cas12a recutting.

Pyr / μM	0	0.98	1.95	3.91	7.81	15.6	31.3	62.5	125	250	500	1000
R106A	1.073	0.576	0.723	0.349	0.074	0.075	0.073	0.066	0.07	0.07	0.068	0.064
	0.966	1.091	0.9	0.541	0.1	0.084	0.071	0.079	0.071	0.069	0.073	0.07
	1.103	0.606	0.833	0.598	0.081	0.08	0.074	0.071	0.072	0.074	0.072	0.07
T107R	1.094	0.527	0.469	0.087	0.071	0.062	0.06	0.068	0.061	0.064	0.068	0.06
	1.047	0.55	0.401	0.124	0.079	0.06	0.064	0.068	0.057	0.066	0.063	0.068
	1.122	0.615	0.555	0.09	0.072	0.064	0.064	0.073	0.066	0.068	0.077	0.064
E236*	1.098	0.789	0.827	0.695	0.171	0.114	0.067	0.064	0.062	0.063	0.071	0.064
	1.049	0.974	1.018	0.866	0.674	0.119	0.067	0.069	0.061	0.07	0.064	0.079
	1.148	0.825	0.958	0.868	0.255	0.153	0.097	0.076	0.07	0.077	0.088	0.071
T107R+E236*	1.146	0.86	1.1	1.014	0.928	0.771	0.16	0.083	0.063	0.061	0.058	0.056
	1.111	1.116	1.1	1.032	0.896	0.723	0.09	0.051	0.057	0.07	0.057	0.063
	1.131	1.079	1.054	1.011	0.918	0.619	0.074	0.039	0.065	0.06	0.057	0.058
S108C	1.2	1.011	0.832	0.757	0.323	0.09	0.086	0.071	0.073	0.084	0.073	0.07
	1.22	0.985	0.787	0.53	0.261	0.107	0.08	0.072	0.075	0.075	0.073	0.076
	1.226	0.781	0.799	0.649	0.124	0.143	0.172	0.073	0.074	0.077	0.073	0.069
S108N	1.512	1.608	1.196	1.633	1.466	1.339	1.048	0.958	0.675	0.142	0.083	0.071
	1.575	1.593	1.556	1.599	1.397	1.36	1.265	1.044	0.868	0.293	0.101	0.078
	1.546	0.988	1.586	1.614	0.885	1.391	1.31	0.917	0.914	0.214	0.091	0.076
WT	1.19	0.166	0.075	0.074	0.075	0.075	0.072	0.08	0.071	0.074	0.068	0.059
	1.146	0.084	0.097	0.077	0.07	0.073	0.07	0.058	0.071	0.062	0.055	0.056
	1.588	0.136	0.086	0.082	0.095	0.106	0.089	0.087	0.075	0.084	0.098	0.084
	1.615	0.092	0.083	0.084	0.065	0.067	0.07	0.059	0.085	0.082	0.068	0.082

Supplementary Figure 16. Pyrimethamine resistance assay of mutant strains, related to figure 5C. For each mutant, three replicates were performed. The number in each well indicates OD600 after extraction of the starting OD600 value (see Methods for details). WT, the wild type *PfDHFR* strain.

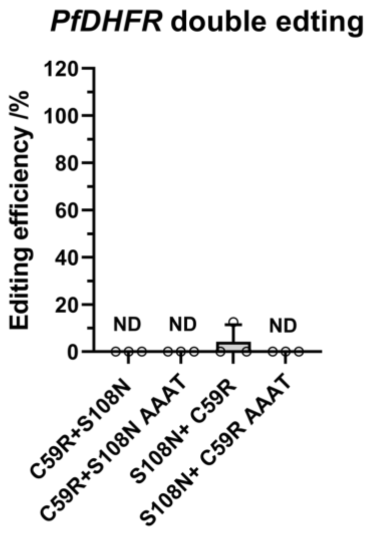

Supplementary Figure 17. The dual editing efficiencies of crRNAs with or without AAAT targeting the non-codon optimized *PfDHFR* gene. Twenty-four transformants of each group were randomly selected for sequencing. ND, not detected. n = 3 biological replicates. Error bars represent standard deviations.

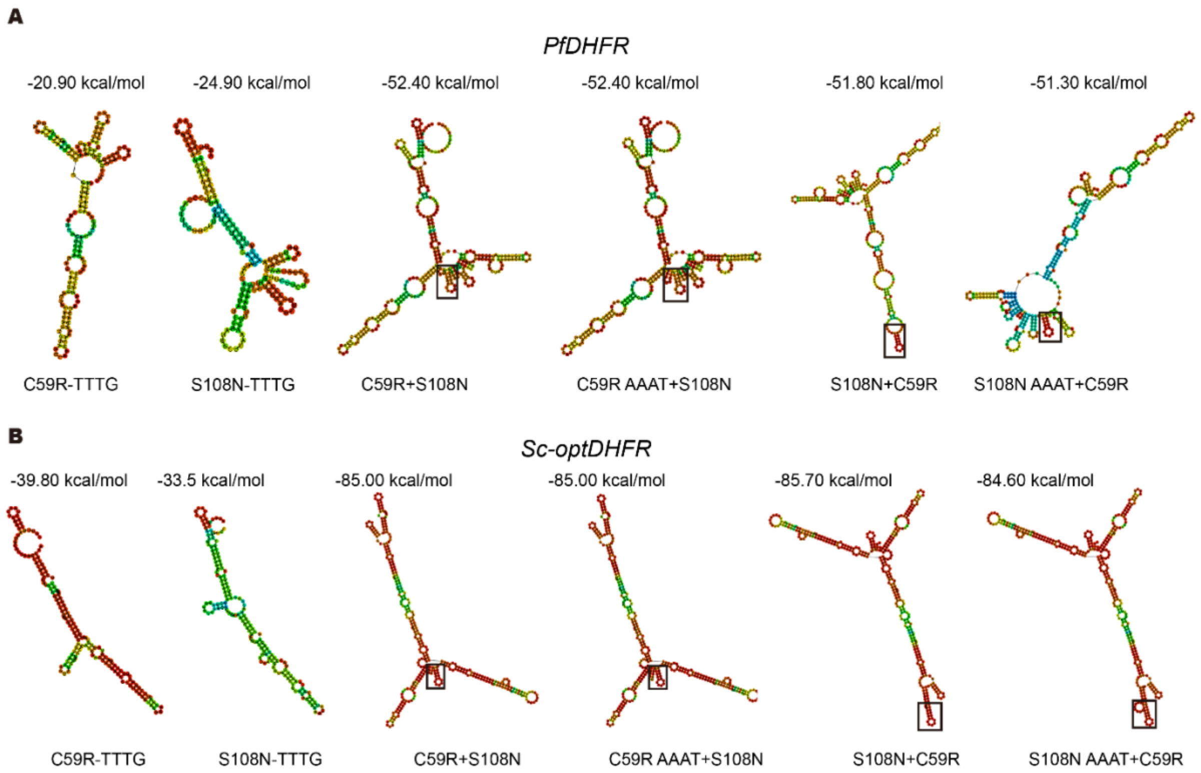

Supplementary Figure 18. (**A**) The predicted secondary structures of single- and duplex-editing crRNA precursors for *PfDHFR*. (**B**) The predicted secondary structures of single- and duplex-editing crRNA precursors for *Sc-optDHFR*. Black rectangles indicate the second DR. RNAfold WebServer (5) was used to predict secondary structures. Minimum free energies of these structures are also shown on top of each structure.

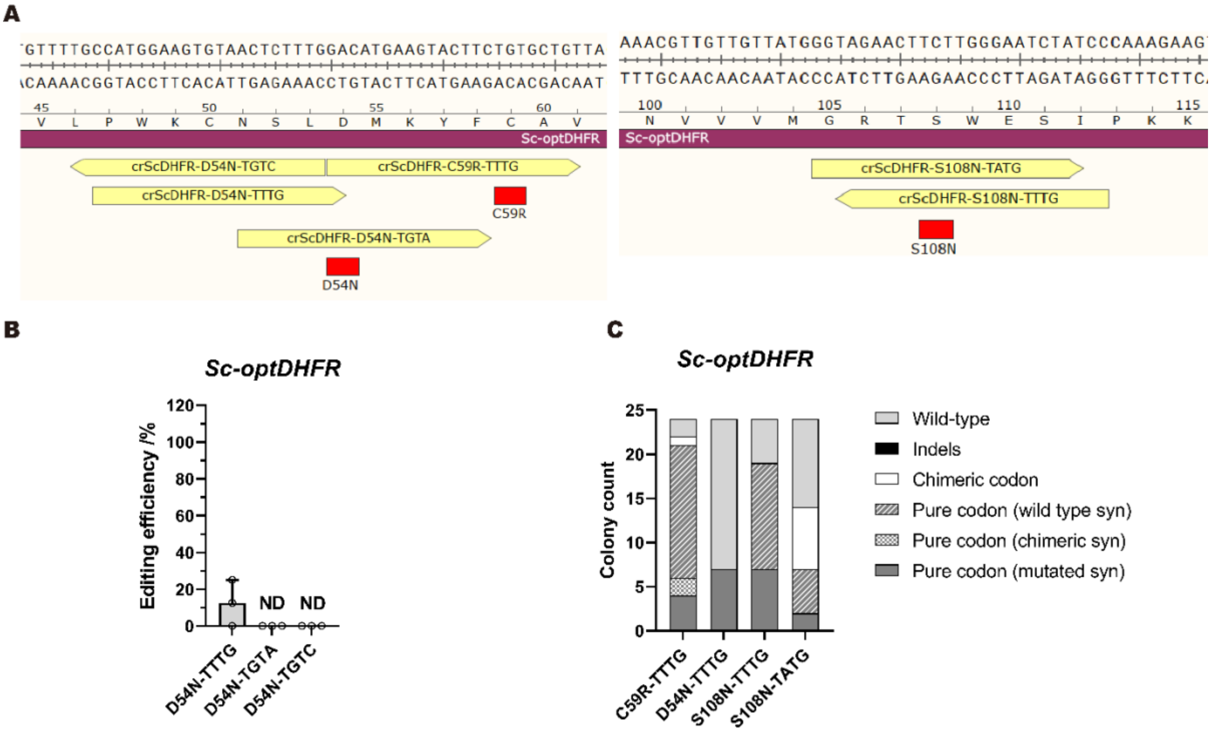

Supplementary Figure 19. (**A**)The spacer sequences of crRNAs used to target *Sc-optDHFR*, related to figure 6B and Supplementary Figure 17B. (**B**) Editing efficiencies assessed by Sanger sequencing of randomly picked colonies. ND, not detected. n = 3 biological replicates. Error bars represent standard deviations. (**C**) The editing accuracy of impLbCas12a targeting *Sc-optDHFR*. syn, synonymous mutations designed to prevent Cas12a recutting.

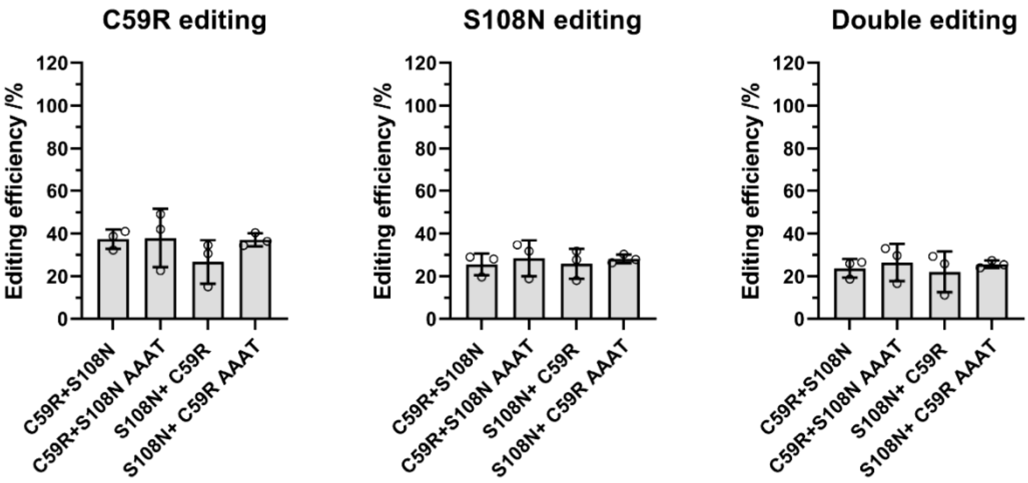

Supplementary Figure 20. The single and dual editing efficiencies of four duplex crRNA arrays targeting *Sc-optDHFR* as assessed by NGS. n = 3 biological replicates. Error bars represent standard deviations.

DNA	counts	Amino Acid	
AGAACAAATTGGGAA	4814291	RTNWE	S108N
AGACGTAGCTGGGAA	718536	RRSWE	T107R
AGACGGAGCTGGGAA	48	RRSWE	
GCGACAAGCTGGGAA	46653	ATSWE	R106A
GCTACAAGCTGGGAA	12	ATSWE	
AGAACATGTTGGGAA	344743	RTCWE	S108C
AGAACAAGCTGGGAA		RTSWE	WT

Supplementary Figure 21. The mutated nucleotide sequences (aa106-110) of *PfDHFR* mutants and their NGS read counts after Pyr selection, related to figure 5B. WT, the wild type *PfDHFR* sequence.

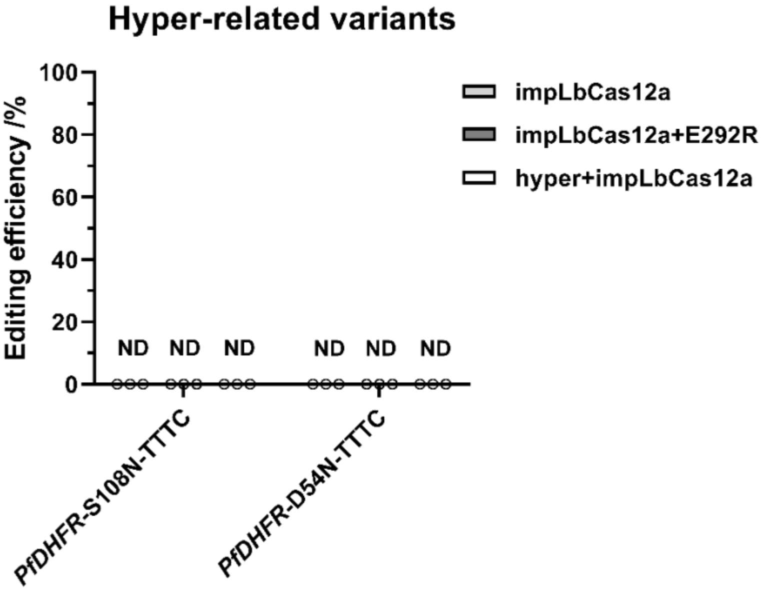

Supplementary Figure 22. The editing efficiencies of hyper-related variants and impLbCas12a with two low-efficiency crRNAs targeting *PfDHFR*. ND, not detected. n = 3 biological replicates.

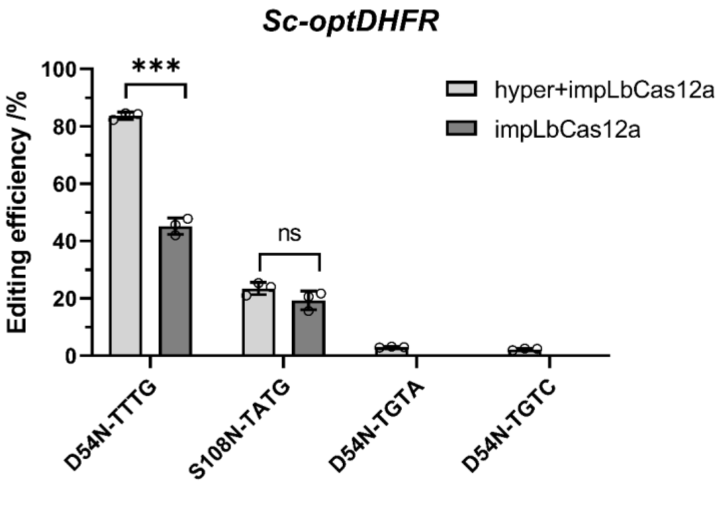

Supplementary Figure 23. Editing efficiencies of hyper+impLbCas12a and impLbCas12a with inefficient crRNAs targeting *Sc-optDHFR* as assessed from NGS data. n = 3 biological replicates. Error bars represent standard deviations. ns, not significant; ***, $P < 0.001$. Significance levels were determined by two-tailed Student's t tests.

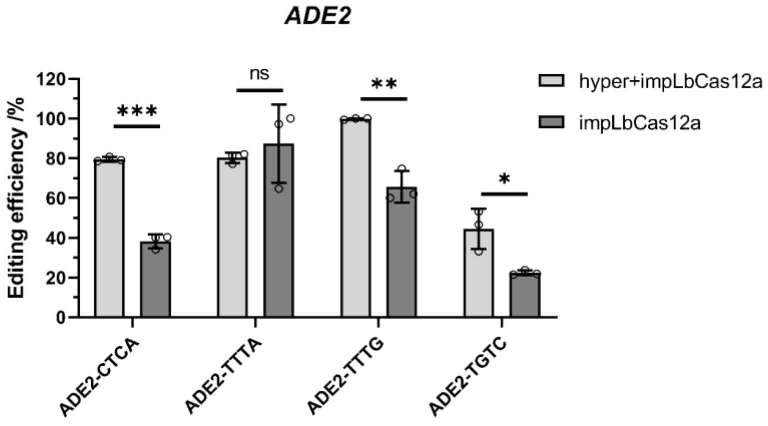

Supplementary Figure 24. Editing efficiency of hyper+impLbCas12a and impLbCas12a targeting *ADE2.* n = 3 biological replicates. Error bars represent standard deviations. ns, not significant; ***, $P < 0.001$, **, $P < 0.01$, *, $P < 0.015$. Significance levels were determined by two-tailed Student's t tests.

SUPPLEMENTARY TABLES

Separate Supplementary Tables

Supplementary Table 1. List of DNA and protein sequences.

Supplementary Table 2. List of primers.

Supplementary Table 3. List of crRNA with donor.

Supplementary Table 4. Cas12a variants with reported and tested PAMs.

Species	Size (aa)	Variant	Reported PAM	Method	Tested PAMs (This study)
Lachnospiraceae bacterium ND2006	1228	LbCas12a-RR	TYYV	In mammalian cells	TCTV
		LbCas12a-RVR	TWTV	In mammalian cells	TATM
		LbCas12a-3Rv	TTYN VTTV TRTV (referring enAsCas12a)	In mammalian cells	VTTG ATTV TATA TGTM
		impLbCas12a	TNTN TACV TTCV TCCV CTCV CCCV	In mammalian cells	TNTN TACC TTCC TCCC CTCM CCCC
Pseudobutyrivibrio ruminis CF1b	1213	PrCas12a-3Rv	VTTV TTCN TCCV TATV	In mammalian cells	NTTG
Francisella novicida U112	1300	FnCas12a-EP16	NNYN NTCA	In vitro	ACTC GGTG CTCA TCTC TCTA GTTG

N=A/T/C/G, V=G/A/T, Y=C/T, W=A/T, M=A/C, R=A/G.

Supplementary Table 5. Mutant distribution in the libraries without and with 4 μM Pyr treatment. * stands for a stop codon. / indicates that corresponding mutant was not found in the NGS data.

Reads	Mutant distribution without Pyr					Mutant distribution with 4 μM Pyr				
	106	107	108	109	110	106	107	108	109	110
*	43	128	310	263	156	1	5	126	12	7
A	21743	7750	8548	/	47842	38908	319	440	/	2153
C	/	246	293928	403	11	/	269	289685	11	9
D	/	12	22	11	8695	/	2	7562	/	363
E	120	14	4	/	485	18	3	3	/	61
F	11	/	95	13548	12	/	/	86	944	42
G	401	42989	526	31	1333	18	1769	120	/	65
H	98	372	/	226	13916	25	354	313	3	353
I	224	101	405	10	4220	7	12	424	/	222
K	69749	278	6	2	99	3030	12	2295	/	13
L	2	337	279	182	28045	/	150	13	7	1410
M	38	37	1	4	12959	7	4	9	/	475
N	36	33	5289	50	31	/	7	4041520	2	26
P	10	51481	798	1	49	/	2252	31	/	6
Q	6	90874	/	458	11105	6	3394	5	/	421
R	3086	480742	1025	115	84	341	601033	523	13	13
S	1107	199346	430546	11	18867	114	8161	19239	4	1026
T	31	59459	190	2	10026	22	2331	362	/	430
V	16	9	27	/	8357	45	1	4	/	476
W	/	9	81	/	7	/	/	66	/	/
Y	4	/	159	154215	680	/	/	912	7843	86

REFERENCES

1. Wang L., Wang H., Liu H., Zhao Q., Liu B., Wang L., Zhang J., Zhu J., Bao R. and Luo Y. 2019. Improved CRISPR-Cas12a-assisted one-pot DNA editing method enables seamless DNA editing. *Biotechnol Bioeng.* 116: 1463-1474.
2. Verwaal R., Buiting-Wiessenhaan N., Dalhuijsen S. and Roubos J.A. 2018. CRISPR/Cpf1 enables fast and simple genome editing of Saccharomyces cerevisiae. *Yeast.* 35: 201-211.
3. Zhu D., Wang J., Yang D., Xi J. and Li J. 2021. High-Throughput Profiling of Cas12a Orthologues and Engineered Variants for Enhanced Genome Editing Activity. *Int J Mol Sci.* 22: 13301.
4. Guo L.Y., Bian J., Davis A.E., Liu P., Kempton H.R., Zhang X., Chemparathy A., Gu B., Lin X., Rane D.A.*, et al.* 2022. Multiplexed genome regulation in vivo with hyper-efficient Cas12a. *Nat Cell Biol.* 24: 590-600.
5. Gruber A.R., Lorenz R., Bernhart S.H., Neuböck R. and Hofacker I.L. 2008. The Vienna RNA websuite. *Nucleic Acids Res.* 36: W70-4.
