# Supplementary material for: Benchmarking the PAM compatibility of Cas12a variants for high-throughput yeast genetic variant engineering
Source: Appl Environ Microbiol. 2025 Nov 25;91(12):e01618-25. doi: 10.1128/aem.01618-25 (PMC12724276; doi:10.1128/aem.01618-25)
Supplement: Table S1 — List of DNA and protein sequences. [file aem.01618-25-s0002.docx]

**Supplementary Table 1. List of DNA and protein sequences**

| pCRCT-impLbCas12a-5’pre-tRNA-crRNA: |
| --- |
| pre-tRNA **DR (from Lb)** Spacer Donor NLS Cas12a  tcgcgcgaacccaactgcacagaacaaaaacctgcaggaaacgaagataaatcatgtcgaaagctacatataaggaacgtgctgctactcatcctagtcctgttgctgccaagctatttaatatcatgcacgaaaagcaaacaaacttgtgtgcttcattggatgttcgtaccaccaaggaattactggagttagttgaagcattaggtcccaaaatttgtttactaaaaacacatgtggatatcttgactgatttttccatggagggcacagttaagccgctaaaggcattatccgccaagtacaattttttactcttcgaagacagaaaatttgctgacattggtaatacagtcaaattgcagtactctgcgggtgtatacagaatagcagaatgggcagacattacgaatgcacacggtgtggtgggcccaggtattgttagcggtttgaagcaggcggcagaagaagtaacaaaggaacctagaggccttttgatgttagcagaattgtcatgcaagggctccctatctactggagaatatactaagggtactgttgacattgcgaagagcgacaaagattttgttatcggctttattgctcaaagagacatgggtggaagagatgaaggttacgattggttgattatgacacccggtgtgggtttagatgacaagggagacgcattgggtcaacagtatagaaccgtggatgatgtcgtttctacaggatctgacattattattgttggaagaggactatttgcaaagggaagggatgctaaggtagagggtgaacgttacagaaaagcaggctgggaagcatatttgagaagatgcggccagcaaaactaaaaaactgtattataagtaaatgcatgtatactaaactcacaaattagagcttcaatttaattatatcagttattaccctgtgctcgaggagctcatagcttcaaaatgtttctactccttttttactcttccagattttctcggactccgcgcatcgccgtaccacttcaaaacacccaagcacagcatactaaatttcccctctttcttcctctagggtgtcgttaattacccgtactaaaggtttggaaaagaaaaaagaggccgcctcgtttctttttcttcgtcgaaaaaggcaataaaaatttttatcacgtttctttttcttgaaaattttttttttgatttttttctctttcgatgacctcccattgatatttaagttaataaacggtcttcaatttctcaagtttcagtttcatttttcttgttctattacaactttttttacttcttgctcattagaaagaaagcatagcaatctaatctaagAACAAAGCACCAGTGGTCTAGTGGTAGAATAGTACCCTGCCACGGTACAGACCCGGGTTCGATTCCCGGCTGGTGCA**AATTTCTACTAAGTGTAGAT**CCGGTTGTGGTATATTTGGTGTGACTCCGTTCAACTTAAGGCGAAGTTGTTGGCAGAAAATGCAATCAAATCTTAACCCGGTTGTGGTATATTTGGTGTGGAAATGTTCTATTTAGAAACAGGGGAATTGCTTATTAACGAAATTcatgtaattagttatgtcacgcttacattcacgccctccccccacatccgctctaaccgaaaaggaaggagttagacaacctgaagtctaggtccctatttatttttttatagttatgttagtattaagaacgttatttatatttcaaatttttcttttttttctgtacagacgcgtgtacgcatgtaacattatactgaaaaccttgcttgagaaggttttgggacgctcgaaggctttaatttgcggccggtaccgcaagtctactagtcagttcgagtttatcattatcaatactgccatttcaaagaatacgtaaataattaatagtagtgattttcctaactttatttagtcaaaaaattagccttttaattctgctgtaacccgtacatgcccaaaatagggggcgggttacacagaatatataacatcgtaggtgtctgggtgaacagtttattcctggcatccactaaatataatggagcccgctttttaagctggcatccagaaaaaaaaagaatcccagcaccaaaatattgttttcttcaccaaccatcagttcataggtccattctcttagcgcaactacagagaacaggggcacaaacaggcaaaaaacgggcacaacctcaatggagtgatgcaacctgcctggagtaaatgatgacacaaggcaattgacccacgcatgtatctatctcattttcttacaccttctattaccttctgctctctctgatttggaaaaagctgaaaaaaaaggttgaaaccagttccctgaaattattcccctacttgactaataagtatataaagacggtaggtattgattgtaattctgtaaatctatttcttaaacttcttaaattctacttttatagttagtcttttttttagttttaaaacaccaagaacttagtttcgaataaacacacataaacaaacaaaacccgccgccagcggccgctaatacgactcactatagggagagccgccaccatgaaacggacagccgacggaagcgagttcgagtcaCCAAAAAAGAAGAGAAAGGTCGGTAGTGGTTCTGGATCCATGAGCAAGCTGGAGAAGTTTACAAACTGCTACTCCCTGTCTAAGACCCTGAGGTTCAAGGCCATCCCTGTGGGCAAGACCCAGGAGAACATCGACAATAAGCGGCTGCTGGTGGAGGACGAGAAGAGAGCCGAGGATTATAAGGGCGTGAAGAAGCTGCTGGATCGCTACTATCTGTCTTTTATCAACGACGTGCTGCACAGCATCAAGCTGAAGAATCTGAACAATTACATCAGCCTGTTCCGGAAGAAAACCAGAACCGAGAAGGAGAATAAGGAGCTGGAGAACCTGGAGATCAATCTGCGGAAGGAGATCGCCAAGGCCTTCAAGGGCAACGAGGGCTACAAGTCCCTGTTTAAGAAGGATATCATCGAGACAATCCTGCCAGAGTTCCTGGACGATAAGGACGAGATCGCCCTGGTGAACAGCTTCAATGGCTTTACCACAGCCTTCACCGGCTTCTTTAGAAACAGAGAGAATATGTTTTCCGAGGAGGCCAAGAGCACATCCATCGCCTTCAGGTGTATCAACGAGAATCTGACCCGCTACATCTCTAATATGGACATCTTCGAGAAGGTGGACGCCATCTTTGATAAGCACGAGGTGCAGGAGATCAAGGAGAAGATCCTGAACAGCGACTATGATGTGGAGGATTTCTTTGAGGGCGAGTTCTTTAACTTTGTGCTGACACAGGAGGGCATCGACGTGTATAACGCCATCATCGGCGGCTTCGTGACCGAGAGCGGCGAGAAGATCAAGGGCCTGAACGAGTACATCAACCTGTATAATCAGAAAACCAAGCAGAAGCTGCCTAAGTTTAAGCCACTGTATAAGCAGGTGCTGAGCGATCGGGAGTCTCTGAGCTTCTACGGCGAGGGCTATACATCCGATGAGGAGGTGCTGGAGGTGTTTAGAAACACCCTGAACAAGAACAGCGAGATCTTCAGCTCCATCAAGAAGCTGGAGAAGCTGTTCAAGAATTTTGACGAGTACTCTAGCGCCGGCATCTTTGTGAAGAACGGCCCCGCCATCAGCACAATCTCCAAGGATATCTTCGGCGAGTGGAACGTGATCCGGGACAAGTGGAATGCCGAGTATGACGATATCCACCTGAAGAAGAAGGCCGTGGTGACCGAGAAGTACGAGGACGATCGGAGAAAGTCCTTCAAGAAGATCGGCTCCTTTTCTCTGGAGCAGCTGCAGGAGTACGCCGACGCCGATCTGTCTGTGGTGGAGAAGCTGAAGGAGATCATCATCCAGAAGGTGGATGAGATCTACAAGGTGTATGGCTCCTCTGAGAAGCTGTTCGACGCCGATTTTGTGCTGGAGAAGAGCCTGAAGAAGAACGACGCCGTGGTGGCCATCATGAAGGACCTGCTGGATTCTGTGAAGAGCTTCGAGAATTACATCAAGGCCTTCTTTGGCGAGGGCAAGGAGACAAACAGGGACGAGTCCTTCTATGGCGATTTTGTGCTGGCCTACGACATCCTGCTGAAGGTGGACCACATCTACGATGCCATCCGCAATTATGTGACCCAGAAGCCCTACTCTAAGGATAAGTTCAAGCTGTATTTTCAGAACCCTCAGTTCATGAGAGGCTGGGACAAGGATGTAGAGACAGACCGTCGGGCCACCATCCTGAGATACGGCTCCAAGTACTATCTGGCCATCATGGATAAGAAGTACGCCAAGTGCCTGCAGAAGATCGACAAGGACGATGTGAACGGCAATTACGAGAAGATCAACTATAAGCTGCTGCCCGGCCCTAATAAGATGCTGCCAAGGGTGTTCTTTTCTAAGAAGTGGATGGCCTACTATAACCCCAGCGAGGACATCCAGAAGATCTACAAGAATGGCACATTCAAGAAGGGCGATATGTTTAACCTGAATGACTGTCACAAGCTGATCGACTTCTTTAAGGATAGCATCTCCCGGTATCCAAAGTGGTCCAATGCCTACGATTTCAACTTTTCTGAGACAGAGAAGTATAAGGACATCGCCGGCTTTTACAGAGAGGTGGAGGAGCAGGGCTATAAGGTGAGCTTCGAGTCTGCCAGCAAGAAGGAGGTGGATAAGCTGGTGGAGGAGGGCAAGCTGTATATGTTCCAGATCTATAACAAGGACTTTTCCGATAAGTCTCACGGCACACCCAATCTGCACACCATGTACTTCAAGCTGCTGTTTGACGAGAACAATCACGGACAGATCAGGCTGAGCGGAGGAGCAGAGCTGTTCATGAGGCGCGCCTCCCTGAAGAAGGAGGAGCTGGTGGTGCACCCAGCCAACTCCCCTATCGCCAACAAGAATCCAGATAATCCCAAGAAAACCACAACCCTGTCCTACGACGTGTATAAGGATAAGAGGTTTTCTGAGGACCAGTACGAGCTGCACATCCCAATCGCCATCAATAAGTGCCCCAAGAACATCTTCAAGATCAATACAGAGGTGCGCGTGCTGCTGAAGCACGACGATAACCCCTATGTGATCGGCATCGATAGGGGCGAGCGCAATCTGCTGTATATCGTGGTGGTGGACGGCAAGGGCAACATCGTGGAGCAGTATTCCCTGAACGAGATCATCAACAACTTCAACGGCATCAGGATCAAGACAGATTACCACTCTCTGCTGGACAAGAAGGAGAAGGAGAGGTTCGAGGCCCGCCAGAACTGGACCTCCATCGAGAATATCAAGGAGCTGAAGGCCGGCTATATCTCTCAGGTGGTGCACAAGATCTGCGAGCTGGTGGAGAAGTACGATGCCGTGATCGCCCTGGAGGACCTGAACTCTGGCTTTAAGAATAGCCGCGTGAAGGTGGAGAAGCAGGTGTATCAGAAGTTCGAGAAGATGCTGATCGATAAGCTGAACTACATGGTGGACAAGAAGTCTAATCCTTGTGCAACAGGCGGCGCCCTGAAGGGCTATCAGATCACCAATAAGTTCGAGAGCTTTAAGTCCATGTCTACCCAGAACGGCTTCATCTTTTACATCCCTGCCTGGCTGACATCCAAGATCGATCCATCTACCGGCTTTGTGAACCTGCTGAAAACCAAGTATACCAGCATCGCCGATTCCAAGAAGTTCATCAGCTCCTTTGACAGGATCATGTACGTGCCCGAGGAGGATCTGTTCGAGTTTGCCCTGGACTATAAGAACTTCTCTCGCACAGACGCCGATTACATCAAGAAGTGGAAGCTGTACTCCTACGGCAACCGGATCAGAATCTTCCGGAATCCTAAGAAGAACAACGTGTTCGACTGGGAGGAGGTGTGCCTGACCAGCGCCTATAAGGAGCTGTTCAACAAGTACGGCATCAATTATCAGCAGGGCGATATCAGAGCCCTGCTGTGCGAGCAGTCCGACAAGGCCTTCTACTCTAGCTTTATGGCCCTGATGAGCCTGATGCTGCAGATGCGGAACAGCATCACAGGCCGCACCGACGTGGATTTTCTGATCAGCCCTGTGAAGAACTCCGACGGCATCTTCTACGATAGCCGGAACTATGAGGCCCAGGAGAATGCCATCCTGCCAAAGAACGCCGACGCCAATGGCGCCTATAACATCGCCAGAAAGGTGCTGTGGGCCATCGGCCAGTTCAAGAAGGCCGAGGACGAGAAGCTGGATAAGGTGAAGATCGCCATCTCTAACAAGGAGTGGCTGGAGTACGCCCAGACCAGCGTGAAGCACAAAAGGCCGGCGGCCACGAAAAAGGCCGGCCAGGCAAAAAAGAAAAAGTAAgtttaaaccgaggcgaatttcttatgatttatgatttttattattaaataagttataaaaaaaataagtgtatacaaattttaaagtgactcttaggttttaaaacgaaaattcttattcttgagtaactctttcctgtaggtcaggttgctttctcaggtatagcatgaggtcgctcttattgaccacacctctaccggcatgccgagcaaatgcctgcaaatcgctccccatttctctagaagcttggcgtaatcatggtcatagctgtttcctgtgtgaaattgttatccgctcacaattccacacaacatacgagccggaagcataaagtgtaaagcctggggtgcctaatgagtgagctaactcacattaattgcgttgcgctcactgcccgctttccagtcgggaaacctgtcgtgccagctgcattaatgaatcggccaacgcgcggggagaggcggtttgcgtattgggcgctcttccgcttcctcgctcactgactcgctgcgctcggtcgttcggctgcggcgagcggtatcagctcactcaaaggcggtaatacggttatccacagaatcaggggataacgcaggaaagaacatgtgagcaaaaggccagcaaaaggccaggaaccgtaaaaaggccgcgttgctggcgtttttccataggctccgcccccctgacgagcatcacaaaaatcgacgctcaagtcagaggtggcgaaacccgacaggactataaagataccaggcgtttccccctggaagctccctcgtgcgctctcctgttccgaccctgccgcttaccggatacctgtccgcctttctcccttcgggaagcgtggcgctttctcatagctcacgctgtaggtatctcagttcggtgtaggtcgttcgctccaagctgggctgtgtgcacgaaccccccgttcagcccgaccgctgcgccttatccggtaactatcgtcttgagtccaacccggtaagacacgacttatcgccactggcagcagccactggtaacaggattagcagagcgaggtatgtaggcggtgctacagagttcttgaagtggtggcctaactacggctacactagaagaacagtatttggtatctgcgctctgctgaagccagttaccttcggaaaaagagttggtagctcttgatccggcaaacaaaccaccgctggtagcggtggtttttttgtttgcaagcagcagattacgcgcagaaaaaaaggatctcaagaagatcctttgatcttttctacggggtctgacgctcagtggaacgaaaactcacgttaagggattttggtcatgagattatcaaaaaggatcttcacctagatccttttaaattaaaaatgaagttttaaatcaatctaaagtatatatgagtaaacttggtctgacagttaccaatgcttaatcagtgaggcacctatctcagcgatctgtctatttcgttcatccatagttgcctgactccccgtcgtgtagataactacgatacgggagggcttaccatctggccccagtgctgcaatgataccgcggctcccacgctcaccggctccagatttatcagcaataaaccagccagccggaagggccgagcgcagaagtggtcctgcaactttatccgcctccatccagtctattaattgttgccgggaagctagagtaagtagttcgccagttaatagtttgcgcaacgttgttgccattgctacaggcatcgtggtgtcacgctcgtcgtttggtatggcttcattcagctccggttcccaacgatcaaggcgagttacatgatcccccatgttgtgcaaaaaagcggttagctccttcggtcctccgatcgttgtcagaagtaagttggccgcagtgttatcactcatggttatggcagcactgcataattctcttactgtcatgccatccgtaagatgcttttctgtgactggtgagtactcaaccaagtcattctgagaatagtgtatgcggcgaccgagttgctcttgcccggcgtcaatacgggataataccgcgccacatagcagaactttaaaagtgctcatcattggaaaacgttcttcggggcgaaaactctcaaggatcttaccgctgttgagatccagttcgatgtaacccactcgtgcacccaactgatcttcagcatcttttactttcaccagcgtttctgggtgagcaaaaacaggaaggcaaaatgccgcaaaaaagggaataagggcgacacggaaatgttgaatactcatactcttcctttttcaatattattgaagcatttatcagggttattgtctcatgagcggatacatatttgaatgtatttagaaaaataaacaaataggggttccgcgcacatttccccgaaaagtgccacctgaacgaagcatctgtgcttcattttgtagaacaaaaatgcaacgcgagagcgctaatttttcaaacaaagaatctgagctgcatttttacagaacagaaatgcaacgcgaaagcgctattttaccaacgaagaatctgtgcttcatttttgtaaaacaaaaatgcaacgcgagagcgctaatttttcaaacaaagaatctgagctgcatttttacagaacagaaatgcaacgcgagagcgctattttaccaacaaagaatctatacttcttttttgttctacaaaaatgcatcccgagagcgctatttttctaacaaagcatcttagattactttttttctcctttgtgcgctctataatgcagtctcttgataactttttgcactgtaggtccgttaaggttagaagaaggctactttggtgtctattttctcttccataaaaaaagcctgactccacttcccgcgtttactgattactagcgaagctgcgggtgcattttttcaagataaaggcatccccgattatattctataccgatgtggattgcgcatactttgtgaacagaaagtgatagcgttgatgattcttcattggtcagaaaattatgaacggtttcttctattttgtctctatatactacgtataggaaatgtttacattttcgtattgttttcgattcactctatgaatagttcttactacaatttttttgtctaaagagtaatactagagataaacataaaaaatgtagaggtcgagtttagatgcaagttcaaggagcgaaaggtggatgggtaggttatatagggatatagcacagagatatatagcaaagagatacttttgagcaatgtttgtggaagcggtattcgcaatattttagtagctcgttacagtccggtgcgtttttggttttttgaaagtgcgtcttcagagcgcttttggttttcaaaagcgctctgaagttcctatactttctagagaataggaacttcggaataggaacttcaaagcgtttccgaaaacgagcgcttccgaaaatgcaacgcgagctgcgcacatacagctcactgttcacgtcgcacctatatctgcgtgttgcctgtatatatatatacatgagaagaacggcatagtgcgtgtttatgcttaaatgcgtacttatatgcgtctatttatgtaggatgaaaggtagtctagtacctcctgtgatattatcccattccatgcggggtatcgtatgcttccttcagcactaccctttagctgttctatatgctgccactcctcaattggattagtctcatccttcaatgctatcatttcctttgatattggatcatctaagaaaccattattatcatgacattaacctataaaaataggcgtatcacgaggccctttcgtc |
| Direct repeat (DR): |
| *Lachnospiraceae bacterium* ND2006 AATTTCTACTAAGTGTAGAT  *Pseudobutyrivibrio ruminis* CF1b AATTTCTACTGTTGTAGAT  *Francisella novicida* AATTTCTACTATGGTAGAT |
| Donor for integration at endogenous promoters (fig. S10): |
| Homology arms (HA) for cloning spacer donor sequence with *Bsa*I sites  GCAAATTTCTACTAAGTgtagattgtactctaaagggttactcctgtactcacaataccaaatgtgaaaaattttagtaacgtaaagtggtttgctATAACAgagaccctttggtctcAAAAAAatgaagcagaaaaaatttaactccaaaaaaagtaatagaacagatttatccatgtaattagttatgtcacg |
| gRNA-*DFR1*∆8bp: |
| Donor spacer  ttgtaggaattgtggcatgtttacagccggagatggggataggatttcgtaccatggaggttgcccagtgaaatgaagtatttcagacaggtcacttcataatggggataggatttcgtgg |
| gRNA-HO spacer: |
| tctgagtcgatcaagccggc |
| gRNA-*DFR1* spacer: |
| acattcattctattgggcag |
| GAPp-*PfDHFR*-ADH1t: |
| HA *PfDHFR*  AGTTTGAATTGCTATTTTATAAGATTCAGGGCCTTCGCCCTTTTTCACAAtcattatcaatactgccatttcaaagaatacgtaaataattaatagtagtgattttcctaactttatttagtcaaaaaattagccttttaattctgctgtaacccgtacatgcccaaaatagggggcgggttacacagaatatataacatcgtaggtgtctgggtgaacagtttattcctggcatccactaaatataatggagcccgctttttaagctggcatccagaaaaaaaaagaatcccagcaccaaaatattgttttcttcaccaaccatcagttcataggtccattctcttagcgcaactacagagaacaggggcacaaacaggcaaaaaacgggcacaacctcaatggagtgatgcaacctgcctggagtaaatgatgacacaaggcaattgacccacgcatgtatctatctcattttcttacaccttctattaccttctgctctctctgatttggaaaaagctgaaaaaaaaggttgaaaccagttccctgaaattattcccctacttgactaataagtatataaagacggtaggtattgattgtaattctgtaaatctatttcttaaacttcttaaattctacttttatagttagtcttttttttagttttaaaacaccaagaacttagtttcgaataaacacacataaacaaacaaaatgctttgcatgataataagagaaattgaagagcgcaacgaactacgagcatgatggaacaagtctgcgacgttttcgatatttatgccatatgtgcatgttgtaaggttgaaagcaaaaatgaggggaaaaaaaatgaggtttttaataactacacatttagaggtctaggaaataaaggagtattaccatggaaatgtaattccctagatatgaaatatttttgtgcagttacaacatatgtgaatgaatcaaaatatgaaaaattgaaatataagagatgtaaatatttaaacaaagaaactgtggataatgtaaatgatatgcctaattctaaaaaattacaaaatgttgtagttatgggaagaacaagctgggaaagcattccaaaaaaatttaaacctttaagcaataggataaatgttatattgtctagaaccttaaaaaaagaagattttgatgaagatgtttatatcattaacaaagttgaagatctaatagttttacttgggaaattaaattactataaatgttttattataggaggttccgttgtttatcaagaatttttagaaaagaaattaataaaaaaaatatattttactagaataaatagtacatatgaatgtgatgtattttttccagaaataaatgaaaatgagtatcaaattatttctgttagcgatgtatatactagtaacaatacaacattggattttatcatttataagaaaacgaataataaaatgttaaatgaacaaaattgtataaaaggagaagaaaaaaataatgatatgcctttaaagaatgatgacaaagatacatgtcatatgaCGGCCGGCttcgaattcactctatacaatcgtaaatgactcgaggcgaatttcttatgatttatgatttttattattaaataagttataaaaaaaataagtgtatacaaattttaaagtgactcttaggttttaaaacgaaaattcttattcttgagtaactctttcctgtaggtcaggttgctttctcaggtatagcatgaggtcgctcttattgaccacacctctaccggTAAGAATGCTTCACGAACTTCTATATGCTCGCCGTACATAAATTCAGGGA |
| *Sc-optDHFR:* |
| HA *Sc-optDHFR*  atgctttgcatgataataagagaaattgaagagcgcaacgaactacgagcATGATGGAACAAGTTTGTGACGTTTTCGACATCTACGCTATCTGTGCTTGTTGTAAGGTTGAATCTAAGAACGAAGGTAAGAAGAACGAAGTTTTCAACAACTACACTTTCAGAGGTTTGGGTAACAAGGGTGTTTTGCCATGGAAGTGTAACTCTTTGGACATGAAGTACTTCTGTGCTGTTACTACTTACGTTAACGAATCTAAGTACGAAAAGTTGAAGTACAAGAGATGTAAGTACTTGAACAAGGAAACTGTTGACAACGTTAACGACATGCCAAACTCTAAGAAGTTGCAAAACGTTGTTGTTATGGGTAGAACTTCTTGGGAATCTATCCCAAAGAAGTTCAAGCCATTGTCTAACAGAATCAACGTTATCTTGTCTAGAACTTTGAAGAAGGAAGACTTCGACGAAGACGTTTACATCATCAACAAGGTTGAAGACTTGATCGTTTTGTTGGGTAAGTTGAACTACTACAAGTGTTTCATCATCGGTGGTTCTGTTGTTTACCAAGAATTCTTGGAAAAGAAGTTGATCAAGAAGATCTACTTCACTAGAATCAACTCTACTTACGAATGTGACGTTTTCTTCCCAGAAATCAACGAAAACGAATACCAAATCATCTCTGTTTCTGACGTTTACACTTCTAACAACACTACTTTGGACTTCATCATCTACAAGAAGACTAACAACAAGATGTTGAACGAACAAAACTGTATCAAGGGTGAAGAAAAGAACAACGACATGCCATTGAAGAACGACGACAAGGACACTTGTCACATGACTGCTGGTTTCGAATTCACTTTGTACAACAGAAAGTAAaacctctccgcccgtatattttttttaatatgttaaatagtgatagaact |
| Protein sequence of *DHFR:* |
| MMEQVCDVFDIYAICACCKVESKNEGKKNEVFNNYTFRGLGNKGVLPWKCNSLDMKYFCAVTTYVNESKYEKLKYKRCKYLNKETVDNVNDMPNSKKLQNVVVMGRTSWESIPKKFKPLSNRINVILSRTLKKEDFDEDVYIINKVEDLIVLLGKLNYYKCFIIGGSVVYQEFLEKKLIKKIYFTRINSTYECDVFFPEINENEYQIISVSDVYTSNNTTLDFIIYKKTNNKMLNEQNCIKGEEKNNDMPLKNDDKDTCHMTAGFEFTLYNRK |
| Duplex crRNA cassette: |
| pre-tRNA **DR (from Lb)** Spacer Donor synSeparators  AACAAAGCACCAGTGGTCTAGTGGTAGAATAGTACCCTGCCACGGTACAGACCCGGGTTCGATTCCCGGCTGGTGCA**AATTTCTACTAAGTGTAGAT**GGATAGATTCCCAAGAAGTTCTAAAGTTCTAGACAAGATAACGTTGATTCTGTTAGACAATGGCTTGAACTTCTTTGGAATAGATTCCCAATTAGTTCTACCCATAACAACAACGTTTTGCAACTTCTTAGAGTTTGGCATGTCGAAAT**AATTTCTACTAAGTGTAGAT**GACATGAAGTACTTCTGTGCTGTACTTTCAGAGGTTTGGGTAACAAGGGTGTTTTGCCATGGAAGTGTAACTCTTTGGATATGAAGTACTTCAGAGCTGTTACTACTTACGTTAACGAATCTAAGTACGAAAAGTTGAAGTACAA |
| Protein sequences of Cas12a variants: |
| *Lachnospiraceae bacterium* ND2006 LbCas12a WT 1228aa  MSKLEKFTNCYSLSKTLRFKAIPVGKTQENIDNKRLLVEDEKRAEDYKGVKKLLDRYYLSFINDVLHSIKLKNLNNYISLFRKKTRTEKENKELENLEINLRKEIAKAFKGNEGYKSLFKKDIIETILPEFLDDKDEIALVNSFNGFTTAFTGFFDNRENMFSEEAKSTSIAFRCINENLTRYISNMDIFEKVDAIFDKHEVQEIKEKILNSDYDVEDFFEGEFFNFVLTQEGIDVYNAIIGGFVTESGEKIKGLNEYINLYNQKTKQKLPKFKPLYKQVLSDRESLSFYGEGYTSDEEVLEVFRNTLNKNSEIFSSIKKLEKLFKNFDEYSSAGIFVKNGPAISTISKDIFGEWNVIRDKWNAEYDDIHLKKKAVVTEKYEDDRRKSFKKIGSFSLEQLQEYADADLSVVEKLKEIIIQKVDEIYKVYGSSEKLFDADFVLEKSLKKNDAVVAIMKDLLDSVKSFENYIKAFFGEGKETNRDESFYGDFVLAYDILLKVDHIYDAIRNYVTQKPYSKDKFKLYFQNPQFMGGWDKDKETDYRATILRYGSKYYLAIMDKKYAKCLQKIDKDDVNGNYEKINYKLLPGPNKMLPKVFFSKKWMAYYNPSEDIQKIYKNGTFKKGDMFNLNDCHKLIDFFKDSISRYPKWSNAYDFNFSETEKYKDIAGFYREVEEQGYKVSFESASKKEVDKLVEEGKLYMFQIYNKDFSDKSHGTPNLHTMYFKLLFDENNHGQIRLSGGAELFMRRASLKKEELVVHPANSPIANKNPDNPKKTTTLSYDVYKDKRFSEDQYELHIPIAINKCPKNIFKINTEVRVLLKHDDNPYVIGIDRGERNLLYIVVVDGKGNIVEQYSLNEIINNFNGIRIKTDYHSLLDKKEKERFEARQNWTSIENIKELKAGYISQVVHKICELVEKYDAVIALEDLNSGFKNSRVKVEKQVYQKFEKMLIDKLNYMVDKKSNPCATGGALKGYQITNKFESFKSMSTQNGFIFYIPAWLTSKIDPSTGFVNLLKTKYTSIADSKKFISSFDRIMYVPEEDLFEFALDYKNFSRTDADYIKKWKLYSYGNRIRIFRNPKKNNVFDWEEVCLTSAYKELFNKYGINYQQGDIRALLCEQSDKAFYSSFMALMSLMLQMRNSITGRTDVDFLISPVKNSDGIFYDSRNYEAQENAILPKNADANGAYNIARKVLWAIGQFKKAEDEKLDKVKIAISNKEWLEYAQTSVKH  impLbCas12a (D156R/G532R/K538V/Y542R/K595R)  MSKLEKFTNCYSLSKTLRFKAIPVGKTQENIDNKRLLVEDEKRAEDYKGVKKLLDRYYLSFINDVLHSIKLKNLNNYISLFRKKTRTEKENKELENLEINLRKEIAKAFKGNEGYKSLFKKDIIETILPEFLDDKDEIALVNSFNGFTTAFTGFFRNRENMFSEEAKSTSIAFRCINENLTRYISNMDIFEKVDAIFDKHEVQEIKEKILNSDYDVEDFFEGEFFNFVLTQEGIDVYNAIIGGFVTESGEKIKGLNEYINLYNQKTKQKLPKFKPLYKQVLSDRESLSFYGEGYTSDEEVLEVFRNTLNKNSEIFSSIKKLEKLFKNFDEYSSAGIFVKNGPAISTISKDIFGEWNVIRDKWNAEYDDIHLKKKAVVTEKYEDDRRKSFKKIGSFSLEQLQEYADADLSVVEKLKEIIIQKVDEIYKVYGSSEKLFDADFVLEKSLKKNDAVVAIMKDLLDSVKSFENYIKAFFGEGKETNRDESFYGDFVLAYDILLKVDHIYDAIRNYVTQKPYSKDKFKLYFQNPQFMRGWDKDVETDRRATILRYGSKYYLAIMDKKYAKCLQKIDKDDVNGNYEKINYKLLPGPNKMLPRVFFSKKWMAYYNPSEDIQKIYKNGTFKKGDMFNLNDCHKLIDFFKDSISRYPKWSNAYDFNFSETEKYKDIAGFYREVEEQGYKVSFESASKKEVDKLVEEGKLYMFQIYNKDFSDKSHGTPNLHTMYFKLLFDENNHGQIRLSGGAELFMRRASLKKEELVVHPANSPIANKNPDNPKKTTTLSYDVYKDKRFSEDQYELHIPIAINKCPKNIFKINTEVRVLLKHDDNPYVIGIDRGERNLLYIVVVDGKGNIVEQYSLNEIINNFNGIRIKTDYHSLLDKKEKERFEARQNWTSIENIKELKAGYISQVVHKICELVEKYDAVIALEDLNSGFKNSRVKVEKQVYQKFEKMLIDKLNYMVDKKSNPCATGGALKGYQITNKFESFKSMSTQNGFIFYIPAWLTSKIDPSTGFVNLLKTKYTSIADSKKFISSFDRIMYVPEEDLFEFALDYKNFSRTDADYIKKWKLYSYGNRIRIFRNPKKNNVFDWEEVCLTSAYKELFNKYGINYQQGDIRALLCEQSDKAFYSSFMALMSLMLQMRNSITGRTDVDFLISPVKNSDGIFYDSRNYEAQENAILPKNADANGAYNIARKVLWAIGQFKKAEDEKLDKVKIAISNKEWLEYAQTSVKH  LbCas12a-RR (G532R/K595R)  MSKLEKFTNCYSLSKTLRFKAIPVGKTQENIDNKRLLVEDEKRAEDYKGVKKLLDRYYLSFINDVLHSIKLKNLNNYISLFRKKTRTEKENKELENLEINLRKEIAKAFKGNEGYKSLFKKDIIETILPEFLDDKDEIALVNSFNGFTTAFTGFFDNRENMFSEEAKSTSIAFRCINENLTRYISNMDIFEKVDAIFDKHEVQEIKEKILNSDYDVEDFFEGEFFNFVLTQEGIDVYNAIIGGFVTESGEKIKGLNEYINLYNQKTKQKLPKFKPLYKQVLSDRESLSFYGEGYTSDEEVLEVFRNTLNKNSEIFSSIKKLEKLFKNFDEYSSAGIFVKNGPAISTISKDIFGEWNVIRDKWNAEYDDIHLKKKAVVTEKYEDDRRKSFKKIGSFSLEQLQEYADADLSVVEKLKEIIIQKVDEIYKVYGSSEKLFDADFVLEKSLKKNDAVVAIMKDLLDSVKSFENYIKAFFGEGKETNRDESFYGDFVLAYDILLKVDHIYDAIRNYVTQKPYSKDKFKLYFQNPQFMRGWDKDKETDYRATILRYGSKYYLAIMDKKYAKCLQKIDKDDVNGNYEKINYKLLPGPNKMLPRVFFSKKWMAYYNPSEDIQKIYKNGTFKKGDMFNLNDCHKLIDFFKDSISRYPKWSNAYDFNFSETEKYKDIAGFYREVEEQGYKVSFESASKKEVDKLVEEGKLYMFQIYNKDFSDKSHGTPNLHTMYFKLLFDENNHGQIRLSGGAELFMRRASLKKEELVVHPANSPIANKNPDNPKKTTTLSYDVYKDKRFSEDQYELHIPIAINKCPKNIFKINTEVRVLLKHDDNPYVIGIDRGERNLLYIVVVDGKGNIVEQYSLNEIINNFNGIRIKTDYHSLLDKKEKERFEARQNWTSIENIKELKAGYISQVVHKICELVEKYDAVIALEDLNSGFKNSRVKVEKQVYQKFEKMLIDKLNYMVDKKSNPCATGGALKGYQITNKFESFKSMSTQNGFIFYIPAWLTSKIDPSTGFVNLLKTKYTSIADSKKFISSFDRIMYVPEEDLFEFALDYKNFSRTDADYIKKWKLYSYGNRIRIFRNPKKNNVFDWEEVCLTSAYKELFNKYGINYQQGDIRALLCEQSDKAFYSSFMALMSLMLQMRNSITGRTDVDFLISPVKNSDGIFYDSRNYEAQENAILPKNADANGAYNIARKVLWAIGQFKKAEDEKLDKVKIAISNKEWLEYAQTSVKH  LbCas12a-RVR (G532R/K538V/Y542R)  MSKLEKFTNCYSLSKTLRFKAIPVGKTQENIDNKRLLVEDEKRAEDYKGVKKLLDRYYLSFINDVLHSIKLKNLNNYISLFRKKTRTEKENKELENLEINLRKEIAKAFKGNEGYKSLFKKDIIETILPEFLDDKDEIALVNSFNGFTTAFTGFFDNRENMFSEEAKSTSIAFRCINENLTRYISNMDIFEKVDAIFDKHEVQEIKEKILNSDYDVEDFFEGEFFNFVLTQEGIDVYNAIIGGFVTESGEKIKGLNEYINLYNQKTKQKLPKFKPLYKQVLSDRESLSFYGEGYTSDEEVLEVFRNTLNKNSEIFSSIKKLEKLFKNFDEYSSAGIFVKNGPAISTISKDIFGEWNVIRDKWNAEYDDIHLKKKAVVTEKYEDDRRKSFKKIGSFSLEQLQEYADADLSVVEKLKEIIIQKVDEIYKVYGSSEKLFDADFVLEKSLKKNDAVVAIMKDLLDSVKSFENYIKAFFGEGKETNRDESFYGDFVLAYDILLKVDHIYDAIRNYVTQKPYSKDKFKLYFQNPQFMRGWDKDVETDRRATILRYGSKYYLAIMDKKYAKCLQKIDKDDVNGNYEKINYKLLPGPNKMLPKVFFSKKWMAYYNPSEDIQKIYKNGTFKKGDMFNLNDCHKLIDFFKDSISRYPKWSNAYDFNFSETEKYKDIAGFYREVEEQGYKVSFESASKKEVDKLVEEGKLYMFQIYNKDFSDKSHGTPNLHTMYFKLLFDENNHGQIRLSGGAELFMRRASLKKEELVVHPANSPIANKNPDNPKKTTTLSYDVYKDKRFSEDQYELHIPIAINKCPKNIFKINTEVRVLLKHDDNPYVIGIDRGERNLLYIVVVDGKGNIVEQYSLNEIINNFNGIRIKTDYHSLLDKKEKERFEARQNWTSIENIKELKAGYISQVVHKICELVEKYDAVIALEDLNSGFKNSRVKVEKQVYQKFEKMLIDKLNYMVDKKSNPCATGGALKGYQITNKFESFKSMSTQNGFIFYIPAWLTSKIDPSTGFVNLLKTKYTSIADSKKFISSFDRIMYVPEEDLFEFALDYKNFSRTDADYIKKWKLYSYGNRIRIFRNPKKNNVFDWEEVCLTSAYKELFNKYGINYQQGDIRALLCEQSDKAFYSSFMALMSLMLQMRNSITGRTDVDFLISPVKNSDGIFYDSRNYEAQENAILPKNADANGAYNIARKVLWAIGQFKKAEDEKLDKVKIAISNKEWLEYAQTSVKH  LbCas12a-3Rv (D156R/G532R/K538R)  MSKLEKFTNCYSLSKTLRFKAIPVGKTQENIDNKRLLVEDEKRAEDYKGVKKLLDRYYLSFINDVLHSIKLKNLNNYISLFRKKTRTEKENKELENLEINLRKEIAKAFKGNEGYKSLFKKDIIETILPEFLDDKDEIALVNSFNGFTTAFTGFFRNRENMFSEEAKSTSIAFRCINENLTRYISNMDIFEKVDAIFDKHEVQEIKEKILNSDYDVEDFFEGEFFNFVLTQEGIDVYNAIIGGFVTESGEKIKGLNEYINLYNQKTKQKLPKFKPLYKQVLSDRESLSFYGEGYTSDEEVLEVFRNTLNKNSEIFSSIKKLEKLFKNFDEYSSAGIFVKNGPAISTISKDIFGEWNVIRDKWNAEYDDIHLKKKAVVTEKYEDDRRKSFKKIGSFSLEQLQEYADADLSVVEKLKEIIIQKVDEIYKVYGSSEKLFDADFVLEKSLKKNDAVVAIMKDLLDSVKSFENYIKAFFGEGKETNRDESFYGDFVLAYDILLKVDHIYDAIRNYVTQKPYSKDKFKLYFQNPQFMRGWDKDRETDYRATILRYGSKYYLAIMDKKYAKCLQKIDKDDVNGNYEKINYKLLPGPNKMLPKVFFSKKWMAYYNPSEDIQKIYKNGTFKKGDMFNLNDCHKLIDFFKDSISRYPKWSNAYDFNFSETEKYKDIAGFYREVEEQGYKVSFESASKKEVDKLVEEGKLYMFQIYNKDFSDKSHGTPNLHTMYFKLLFDENNHGQIRLSGGAELFMRRASLKKEELVVHPANSPIANKNPDNPKKTTTLSYDVYKDKRFSEDQYELHIPIAINKCPKNIFKINTEVRVLLKHDDNPYVIGIDRGERNLLYIVVVDGKGNIVEQYSLNEIINNFNGIRIKTDYHSLLDKKEKERFEARQNWTSIENIKELKAGYISQVVHKICELVEKYDAVIALEDLNSGFKNSRVKVEKQVYQKFEKMLIDKLNYMVDKKSNPCATGGALKGYQITNKFESFKSMSTQNGFIFYIPAWLTSKIDPSTGFVNLLKTKYTSIADSKKFISSFDRIMYVPEEDLFEFALDYKNFSRTDADYIKKWKLYSYGNRIRIFRNPKKNNVFDWEEVCLTSAYKELFNKYGINYQQGDIRALLCEQSDKAFYSSFMALMSLMLQMRNSITGRTDVDFLISPVKNSDGIFYDSRNYEAQENAILPKNADANGAYNIARKVLWAIGQFKKAEDEKLDKVKIAISNKEWLEYAQTSVKH  Hyper+impLbCas12a (D156R/D235R/E292R/D350R/G532R/K538V/Y542R/K595R)  MSKLEKFTNCYSLSKTLRFKAIPVGKTQENIDNKRLLVEDEKRAEDYKGVKKLLDRYYLSFINDVLHSIKLKNLNNYISLFRKKTRTEKENKELENLEINLRKEIAKAFKGNEGYKSLFKKDIIETILPEFLDDKDEIALVNSFNGFTTAFTGFFRNRENMFSEEAKSTSIAFRCINENLTRYISNMDIFEKVDAIFDKHEVQEIKEKILNSDYDVEDFFEGEFFNFVLTQEGIRVYNAIIGGFVTESGEKIKGLNEYINLYNQKTKQKLPKFKPLYKQVLSDRESLSFYGRGYTSDEEVLEVFRNTLNKNSEIFSSIKKLEKLFKNFDEYSSAGIFVKNGPAISTISKRIFGEWNVIRDKWNAEYDDIHLKKKAVVTEKYEDDRRKSFKKIGSFSLEQLQEYADADLSVVEKLKEIIIQKVDEIYKVYGSSEKLFDADFVLEKSLKKNDAVVAIMKDLLDSVKSFENYIKAFFGEGKETNRDESFYGDFVLAYDILLKVDHIYDAIRNYVTQKPYSKDKFKLYFQNPQFMRGWDKDVETDRRATILRYGSKYYLAIMDKKYAKCLQKIDKDDVNGNYEKINYKLLPGPNKMLPRVFFSKKWMAYYNPSEDIQKIYKNGTFKKGDMFNLNDCHKLIDFFKDSISRYPKWSNAYDFNFSETEKYKDIAGFYREVEEQGYKVSFESASKKEVDKLVEEGKLYMFQIYNKDFSDKSHGTPNLHTMYFKLLFDENNHGQIRLSGGAELFMRRASLKKEELVVHPANSPIANKNPDNPKKTTTLSYDVYKDKRFSEDQYELHIPIAINKCPKNIFKINTEVRVLLKHDDNPYVIGIDRGERNLLYIVVVDGKGNIVEQYSLNEIINNFNGIRIKTDYHSLLDKKEKERFEARQNWTSIENIKELKAGYISQVVHKICELVEKYDAVIALEDLNSGFKNSRVKVEKQVYQKFEKMLIDKLNYMVDKKSNPCATGGALKGYQITNKFESFKSMSTQNGFIFYIPAWLTSKIDPSTGFVNLLKTKYTSIADSKKFISSFDRIMYVPEEDLFEFALDYKNFSRTDADYIKKWKLYSYGNRIRIFRNPKKNNVFDWEEVCLTSAYKELFNKYGINYQQGDIRALLCEQSDKAFYSSFMALMSLMLQMRNSITGRTDVDFLISPVKNSDGIFYDSRNYEAQENAILPKNADANGAYNIARKVLWAIGQFKKAEDEKLDKVKIAISNKEWLEYAQTSVKH  *Pseudobutyrivibrio ruminis* CF1b PrCas12a-3Rv (E162R/N519R/K525R)  MIIGRDFNMYYqnltkmypisktlrnelipvgktlenirkngileadiqrkadyehvkklmdnyhkqlinealqgvhlsdlsdaydlyfnlskeknsvdafskcqdklrkeivsllknhenfpkignkeiikllqslydndtdykaldsfsnfytyfssynRvrknlysdeeksstvayrlinenlpkfldnikayaiakkagvraeglseedqdclfiietfertltqdgidnynaaigklntainlfnqqnkkqegfrkvpqmkclykqilsdreeafidefsddedlitniesfaenmnvflnseiitdfkialvesdgslvyikndvsktsfsnivfgswnaideklsdeydlanskkkkdekyyekrqkelkknksydletiiglfddnsdvigkyieklesditaiaeakndfdeivlrkhdknkslrkntnaveaiksyldtvkdferdiklingsgqeveknlvvyaeqenilaeiknvdslynmsrnyltqkpfstekfklnfnratllRgwdknRetdnlgilfekdgmyylgimntkankifvnipkatsndvyhkvnykllpgpnkmlpkvffaqsnldyykpseellakykagthkkgdnfsledchalidffkasiekhpdwssfgfefsetctyedlsgfyrevekqgykitytdvdadyitslverdelylfqiynkdfspyskgnlnlhtiylqmlfdqrnlnnvvyklngeaevfyrpasindeeviihkageeiknknskravdkptskfgydiikdrryskdkfmlhipvtmnfgvdetrrfndvvndalrndekvrvigidrgernllyvvvvdtdgtileqislnsiinneysietdyhklldekegdrdrarknwttienikelkegylsqvvnviaklvlkynaiicledlnfgfkrgrqkvekqvyqkfekmlidklnylvidksrkqdkpeefggalnalqltskftsfkdmgkqtgiiyyvpayltskidpttgfanlfyvkyenvekakeffsrfdsisynnesgyfefafdykkftdracgarsqwtvctygeriikfrnteknnsfddktivlseefkelfsiygisyedgaelknkimsvdeadffrsltrlfqqtmqmrnssndvtrdyiispimndrgeffnseacdaskpkdadangafniarkglwvleqirntpsgdklnlamsnaewleyaqrnqi  *Francisella novicida* FnCas12a-EP16 (K180S/N607R/K613V/D616N/N617R/K660R)  MSIYQEFVNKYSLSKTLRFELIPQGKTLENIKARGLILDDEKRAKDYKKAKQIIDKYHQFFIEEILSSVCISEDLLQNYSDVYFKLKKSDDDNLQKDFKSAKDTIKKQISEYIKDSEKFKNLFNQNLIDAKKGQESDLILWLKQSKDNGIELFKANSDITDIDEALEIIKSFKGWTTYFSGFHENRKNVYSSNDIPTSIIYRIVDDNLPKFLENKAKYESLKDKAPEAINYEQIKKDLAEELTFDIDYKTSEVNQRVFSLDEVFEIANFNNYLNQSGITKFNTIIGGKFVNGENTKRKGINEYINLYSQQINDKTLKKYKMSVLFKQILSDTESKSFVIDKLEDDSDVVTTMQSFYEQIAAFKTVEEKSIKETLSLLFDDLKAQKLDLSKIYFKNDKSLTDLSQQVFDDYSVIGTAVLEYITQQIAPKNLDNPSKKEQELIAKKTEKAKYLSLETIKLALEEFNKHRDIDKQCRFEEILANFAAIPMIFDEIAQNKDNLAQISIKYQNQGKKDLLQASAEDDVKAIKDLLDQTNNLLHKLKIFHISQSEDKANILDKDEHFYLVFEECYFELANIVPLYNKIRNYITQKPYSDEKFKLNFENSTLARGWDKNVEPNRTAILFIKDDKYYLGVMNKKNNKIFDDKAIKENKGEGYKKIVYRLLPGANKMLPKVFFSAKSIKFYNPSEDILRIRNHSTHTKNGSPQKGYEKFEFNIEDCRKFIDFYKQSISKHPEWKDFGFRFSDTQRYNSIDEFYREVENQGYKLTFENISESYIDSVVNQGKLYLFQIYNKDFSAYSKGRPNLHTLYWKALFDERNLQDVVYKLNGEAELFYRKQSIPKKITHPAKEAIANKNKDNPKKESVFEYDLIKDKRFTEDKFFFHCPITINFKSSGANKFNDEINLLLKEKANDVHILSIDRGERHLAYYTLVDGKGNIIKQDTFNIIGNDRMKTNYHDKLAAIEKDRDSARKDWKKINNIKEMKEGYLSQVVHEIAKLVIEYNAIVVFEDLNFGFKRGRFKVEKQVYQKLEKMLIEKLNYLVFKDNEFDKTGGVLRAYQLTAPFETFKKMGKQTGIIYYVPAGFTSKICPVTGFVNQLYPKYESVSKSQEFFSKFDKICYNLDKGYFEFSFDYKNFGDKAAKGKWTIASFGSRLINFRNSDKNHNWDTREVYPTKELEKLLKDYSIEYGHGECIKAAICGESDKKFFAKLTSVLNTILQMRNSKTGTELDYLISPVADVNGNFFDSRQAPKNMPQDADANGAYHIGLKGLMLLGRIKNNQEGKKLNLVIKNEEYFEFVQNRNN |
